# Predicting Future Performance in Powerlifting: A Machine Learning Approach

**DOI:** 10.1186/s40798-025-00903-z

**Published:** 2025-10-01

**Authors:** Luca Ferrari, Gianluca Bochicchio, Alberto Bottari, Francesco Lucertini, Silvia Pogliaghi

**Affiliations:** 1 Department of Neurosciences, Biomedicine and Movement Sciences, University of Verona, Verona, 37131 Italy; 2 Department of Biomolecular Sciences, University of Urbino, Urbino, 61029 Italy; 3 University of Western Ontario, Research Associate Canadian Center for Activity and Ageing, London, ON N6A 3K7 Canada

## Abstract

**Background:**

Powerlifting is a discipline in which athletes aim to lift the maximum weight in 3 exercises: Squat, Bench Press, and Deadlift. Since the introduction of “Classic” powerlifting by the International Powerlifting Federation (IPF) in 2012, there has been an increase in popularity, athlete participation, and attention from sports science research. Previous studies have examined factors influencing the long-term longitudinal adaptation of upper- and lower-body strength, but no one used this information to develop predictive models of future classic powerlifting performances, especially considering the different age, sex, and weight categories, with the final aim of tailoring the medium- and long-term training goals. This study aims to develop and validate a machine learning-based linear regression model to predict single-lift and overall performance in classic powerlifters. The model considered variables such as sex, age, weight, initial strength levels, and competition history. The study also seeks to provide European normative powerlifting performance data across different categories to assist in talent identification and optimization of training.

**Results:**

The final dataset included 54,064 observations from 8,907 unique lifters. Normative data differed between sex, age categories, and initial strength level (*p* < 0.001). The predictive model demonstrated high predictive accuracy (Root mean Square of Error 10.41 to 19.4; R^2^ 0.90 to 0.94), with no differences between mean values (p 0.733 to 0.930), extremely large correlations (r 0.95 to 0.97), and no significant bias (z-score − 1.78 to − 0.64) between predicted and actual performance values across all lifts.

**Conclusions:**

The developed machine learning model provides valid and accurate predictions of individual powerlifting performance, by accounting for various individual characteristics. The model can assist coaches and athletes in setting realistic training goals and monitoring progress. Moreover, normative data for each lift and total performance were provided, stratified by sex, age, weight category, and initial strength levels, offering valuable benchmarks for athletes and coaches.

**Supplementary Information:**

The online version contains supplementary material available at 10.1186/s40798-025-00903-z.

## Key Points

A machine learning approach could be used to develop a quantitative predictive model of performance trajectory based on individual characteristics that could serve as a tool for personalizing the medium- and long-term training goals.

The powerlifting normative data provided across age span and between sexes could support athletes and coaches in detecting individual lift deficiencies or be used for talent identification and stratification.

Longitudinal data from powerlifting athletes provide valuable insights into the protective effects against age-related decline in muscle strength in older individuals, peak strength development in youth, and the participation of females individuals in strength and power sports.

**Supplementary Information:**

The online version contains supplementary material available at 10.1186/s40798-025-00903-z.

## Background

Powerlifting (PL) is a discipline in which athletes aim to lift the maximum weight in 3 exercises: Squat, Bench Press, and Deadlift. In 2012, the International Powerlifting Federation held the first “Classic” competition in which no supportive equipment for lifting was admitted (i.e. only a singlet, wrist wraps, knee sleeves, and belt admitted). The executions of the lifts have to respect the standards of the International Powerlifting Federation (IPF) [1] and athletes compete into weight, age, and sex categories [2]. Since 2012, this discipline has increased in popularity, athlete participation [3], and attention from sports science research. Indeed, different aspects of powerlifting performance, from the biomechanics of the lifting technique [4–7] to anthropometrics [8–10] and performance determinants [11, 12], have been investigated, to guide athletes’ selection and training practices towards performance optimization.

In this context, recent studies described the factors influencing the long-term longitudinal adaptation of upper- and lower-body strength, the main determinant in the powerlifting performance. In particular, Latella et al. [13] used a retrospective analysis of 15 years of powerlifting competitions to investigate the effect of weight, sex, and age categories on long-term strength adaptation. The authors found that along with the effect of time (i.e., experience or career duration), weight, sex, and age were all important variables affecting long-term strength adaptations. In particular, the increase in strength for both sexes was greatest in the earliest phase of PL participation ( ~ + 7.5–12.5% in the first year, then slowly reaching ~ + 20% in the tenth year); moreover, adult female powerlifters displayed faster progression than males (10-12.5% vs. 5-7.5% in the first year). Interestingly, while a slight strength loss was observed in Master 4 (> 69 years) male athletes (~ 0.35%/year), Master females (Master 3 > 59 years and Master 4 > 69 years) showed a ~ 0.21–0.33%/year strength improvement over ~ 15 years. Finally, among the factors that appear to affect the rate of long-term strength adaptation is the baseline strength at the beginning of the powerlifting career [3, 14]. Indeed, the strongest males displayed a lower improvement rate of strength than the least strong males (0.102 ± 0.330 vs. 0.211 ± 0.355 strength gain (kg)/day). Interestingly, strength gains in females over a 15-year period did not significantly differ between strongest vs. least strong athletes (0.097 ± 0.109 vs. 0.168 ± 1.25 strength gain (kg) /day), though with a very high between individuals variability [14].

Machine learning is a sub-type of artificial intelligence that develops algorithms that are able to identify patterns in data [15]. This technique has recently been used in sports sciences, particularly for injury, competition success odds, and quantitative performance prediction in sports [12, 15–19]. These predictive models could be used as a tool for helping coaches and athletes in the decision-making process, tailoring training plan, assessing injury risk, and monitoring performances. A machine learning approach could be used to develop a quantitative predictive model of performance trajectory based on individual characteristics that could serve as a tool for personalizing the medium- and long-term training goals. In this context, a machine learning approach was used in the Powerlifting discipline to predict future performance [20]. Chau et al. retrospectively analyzed powerlifting records from 1972 to 2017 with an “unsupervised” method of machine learning called the Extreme Learning Machine (ELM) for powerlifting best score prediction [20]. The above study represents the first step towards the quantitative prediction of performance in powerlifting, despite it did not consider female athletes, included both equipped and classic powerlifting, did not predict strength changes over time, and was developed with a hardly interpretable method (i.e., the “black box” method). A further improvement in this approach should consider the development of an interpretable machine learning model able to predict strength changes over time, including female powerlifting athletes.

In this context, linear and logistic regression, a “supervised” Machine learning subtype [21–23], by relating one or more individual variables to some outcome based on associations observed in a large dataset may offer a more comprehensible (i.e. easier interpretation of linear model coefficients over other regression models) [24] classification and prediction tool. Moreover, the inclusion of data from both male and female athletes of different age categories of classic-only powerlifting could offer a more sport-specific, accurate, and generalizable tool compared to the previously developed ELM model.

OpenPowerlifting [25] is a permanent, open-access archive, which contains continuously updated worldwide powerlifting data from 1974 to today. The availability of this large historical data, with 49 years of powerlifting competition results, offers the opportunity to explore the prediction ability of a supervised machine learning model that implements the classic powerlifters’ individual, historical, and anthropometric data.

Cultural, social, and economic factors of different geographic areas could affect powerlifting popularity and diffusion, which in turn could affect training practices, competition levels, and discipline developments [26]. In this context, data regarding longitudinal strength development in Classic European powerlifters is lacking as well as a comprehensive and detailed description of European normative powerlifting performance data.

This study aims to develop and validate a comprehensive linear regression machine learning model for the prediction of single lifts and overall performance in powerlifters of both sexes, across different age and weight categories and initial strength levels and within the relatively homogeneous population and cultural context of the European nations. This model could help athletes and coaches plan realistic goals and monitor the individual’s adherence to optimal performance trajectory over time. Moreover, the availability of normative data could help coaches in talent identification and training-goal setting by comparing talents’ strength levels against normative values of competitors with similar age and weight categories.

We hypothesized that the model developed with supervised artificial intelligence will accurately predict single lifts and overall performances in powerlifters of both sexes, different age and weight categories, and initial strength levels.

## Methods

### Study Design

This study used a public domain, free-use, large data set of powerlifting performance records [25]. The retrospective analysis of historical performance data was used to model and predict future performances. Given the nature of the data used in this study, the University Ethical Board waived the requirement for ethics approval and informed consent to participate, and all methods were carried out in accordance with University of Verona ethical board regulations.

### Data Preparation

The available complete database of historical powerlifting records includes 1 251 502 entries, relative to 310 562 unique lifters, of which 119 055 (38.3%) were females and 191 480 (61.7%) males. Out of these, we considered only data from 2012 to 2024. The above were further filtered to include only classic powerlifting events and to include only the European powerlifting federations affiliated with the International Powerlifting Federation (IPF). The resultant dataset was filtered again to include data from complete (in which all three exercises were performed) events only. From this newly made database, we manually excluded any lifter that had competed in only one competition (lack of longitudinal information). In addition, if an observation was incomplete (i.e., a lifter failed all attempts for a given lift and was therefore disqualified), the respective row was deleted [13]. Each athlete’s performance was computed as the best of all attempts for squat, bench press, deadlift, and total (sum of best attempts across all three lifts) for each competition.

The filtered database contained, for each athlete and each one of the competitions attended, data relative to the date of the competition, the athlete’s sex, age, weight category, body weight, and best performance in all lifts and total. To account for the individual initial strength level, relative strength was computed by dividing the first absolute performance by the respective athletes’ body weight, for each lift. Moreover, for each competition attended by the same participant, the time from the first competition was computed in weeks and log-transformed by computing its natural logarithm (Log_time) to allow a linear description of performance data [13]. To provide powerlifting performance normative data, athletes were stratified in Quartiles (Q1, Q2, Q3, Q4) based on initial strength level, independently for each lift (Total, Squat, Bench Press, and Deadlift), age (Sub-junior ≤ 18 years, Junior > 18 ≤ 23 years, Open > 23 ≤ 39 years, Masters 1 > 39 ≤ 49 years, Masters 2 > 49 ≤ 59 years, Masters 3 > 59 ≤ 69 years, and Masters 4 > 69 years), body weight (Females: 47 kg, 52 kg, 57 kg, 63 kg, 69 kg, 76 kg, 84 kg, and > 84 kg; males: 59 kg, 66 kg, 74 kg, 83 kg, 93 kg, 105 kg, 120 kg, and > 120 kg.), and sex categories (Males and Females) [27]. Q1 identified the weakest athletes in the bottom 25th percentile, Q2 and Q3 identified athletes within the 25 and 50th and the 50 to 75th percentile, respectively, while Q4 identified the strongest athletes in the > 75th percentile.

### Development of the AI Based Prediction Model (training the Model)

All the analyses were computed using Matlab (Version R2021B, MathWorks Inc, Natick, Massachusetts, USA).

A small random sample of 100 athletes was used to preliminary analyze the best machine learning approach. Different machine learning methods, including decision tree, ensemble method, and linear regression models, were trained on this sample. The linear regression model displayed the best data fit. The performance of the above modeling strategies is presented in the supplementary materials.

The whole data set was then linear fitted using the “regression learner tool” in Matlab, selecting the following scalar and continuous input variables: sex category, age, initial strength level, body weight, and Log_time, while the absolute strength (for Squat, Bench Press, Deadlift, and Total) was selected as the outcome variable. The holdout validation method was used to test the prediction model’s validity [28]. Briefly, this method randomly selects a percentage of the data for model training while the remaining part is used for testing model performance. For the purpose of this study, 80% of the database was used to train the prediction model, and the remaining 20% was used to test the model performance. The holdout method was chosen among others (i.e., leave-one-out or k-fold methods) since it is free from overfitting and is generally used with larger data sets like ours. Therefore, a single random subdivision of the data set is sufficient to validate the model [29]. In the supplementary materials, the comparison between our model validated with the hold-out and k-fold methods for the Total score is reported. The model equation formula expressed in its explicit form was the following:

$$\begin{aligned}&Pr={\beta\:}_{0}+{\beta\:}_{1}{X}_{sex}+{\beta\:}_{2}{X}_{age}+{\beta\:}_{3}{X}_{bodyweight}\\&+{\beta\:}_{4}{X}_{initial\:strength}\\&+{\beta\:}_{5}{X}_{log\_time}\\&+{\beta\:}_{6}{X}_{sex*age}\\&+{\beta\:}_{7}{X}_{sex*bodyweight}\\&+{\beta\:}_{8}{X}_{sex*initial\:strength}\\&+{\beta\:}_{9}{X}_{sex*log\_time}+{\beta\:}_{10}{X}_{age*bodyweight}\\&+{\beta\:}_{11}{X}_{age*initial\:strength}\\&+{\beta\:}_{12}{X}_{age*log\_time}\\&+{\beta\:}_{13}{X}_{bodyweight*initial\:strength}\\&+{\beta\:}_{14}{X}_{bodyweight*log\_time}\\&+{\beta\:}_{15}{X}_{initial\:strength*log}\end{aligned}$$

Where Pr is the absolute strength (in each lift and total, separately), β0 is the intercept, *βi* are the coefficients, *X*_*sex*_ is the indicator variable for Sex (Males = 0; Females = 1), *X*_*age*_ is the Age variable (years), *X*_*bodyweight*_ is the Bodyweight variable (kg), *X*_*initial strength*_ is the Initial Strength Level variable (kg/kg), and *Xlog_time*is the Log_time variable.

### Statistical Analysis

Descriptive statistics were calculated and reported as mean ± standard deviation. A three-way ANOVA was run to independently compare athletes’ initial strength levels between sex, age categories, and quartiles.

The predictive validity of the developed model equation was assessed in 20% of the data set, corresponding to 10.812 entries. Mean Absolute Error (MAE), Mean Squared Error (MSE), Root Mean Squared Error (RMSE), and Coefficient of Determination (R-squared) were calculated in this subset independently for each lift and total. Actual and predicted absolute performance values for each lift and total were compared by 2 way ANOVA (sex x method) and Pearson’s correlation coefficient. Moreover, a Bland–Altman analysis [30] was run to compute the bias, precision, and limits of agreement (LOA) between actual and predicted values. Then, a one-sided z-test on the bias was run. The correlation coefficient (r) was interpreted as follows: trivial (< 0.1); small (0.10–0.29); moderate (0.30–0.49); large (0.50–0.69); very large (0.70–0.89); extremely large (0.90–1.00) [31]. The significance level was set at *p* < 0.05.

## Results

**Fig. 1:**
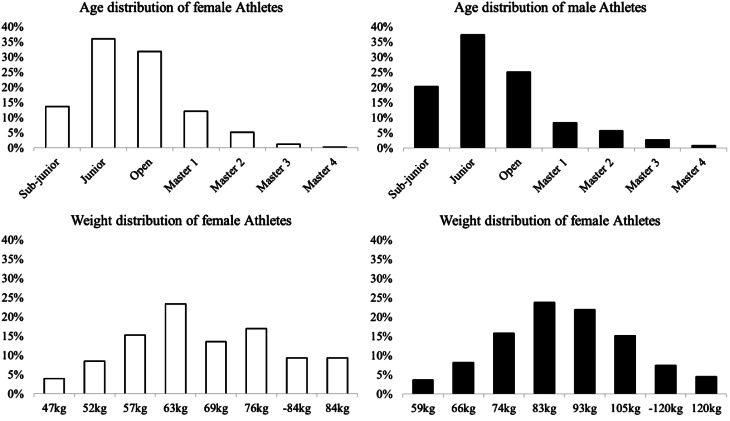
The percentage distribution of female (white columns) and male (black columns) powerlifter athletes are represented in their weight and age categories. Sub-junior identifies athletes ≤ 18 years, Junior > 18 ≤ 23 years, Open > 23 ≤ 39 years, Masters 1 > 39 ≤ 49 years, Masters 2 > 49 ≤ 59 years, Masters 3 > 59 ≤ 69 years, and Masters 4 > 69 years. Females and Males athletes are divided in weight categories and expressed in kilograms (Females: 47 kg, 52 kg, 57 kg, 63 kg, 69 kg, 76 kg, 84 kg, and > 84 kg; males: 59 kg, 66 kg, 74 kg, 83 kg, 93 kg, 105 kg, 120 kg, and > 120 kg). “− ± −“ indicates a lack of data to compute the mean ± Standard deviation. The Junior and Open categories displayed the largest participation of athletes of both sexes. The middle-weight categories of both sexes displayed the largest participation of athletes

The final database contained 54,064 observations relative to 8907 unique lifters, of which 3183 (35.7%) were females and 5724 (64.3%) were males. The distributions of female and male athletes in their weight and age categories are presented in Fig. 1.

The mean time from the first was 149.24 ± 120.94 weeks (3.11 ± 2.52 years) and 168.34 ± 105.41 weeks (3.51 ± 2.19 years) for females and males, respectively. Average, measured initial strength level data in Squats, Bench Presses, Deadlifts, and Total relative to sex, body weight, and age categories for each quartile are reported in Table 1a, 1b, 1c, 1d, respectively.

**Table 1:** **A**: Squat normative data for European powerlifting athletes divided by sex, age, weight categories, and quartiles. Data are presented as mean ± standard deviation

	Males									Females										
	Squat																	Main Effect		
	59 kg	66 kg	74 kg	83 kg	93 kg	105 kg	-120 kg	120 kg		47 kg	52 kg	57 kg	63 kg	69 kg	76 kg	-84 kg	84 kg	Sex	Age cat.	Quartile
Sub-junior																		< 0.001	< 0.001	< 0.001
Q1	1.44 ± 0.27	1.56 ± 0.22	1.59 ± 0.17	1.60 ± 0.15	1.58 ± 0.20	1.56 ± 0.17	1.55 ± 0.18	1.41 ± 0.29		1.17 ± 0.17	1.20 ± 0.09	1.09 ± 0.14	1.17 ± 0.11	1.11 ± 0.11	1.18 ± 0.15	1.20 ± 0.14	1.09 ± 0.14			
Q2	1.92 ± 0.07	1.94 ± 0.06	1.92 ± 0.06	1.92 ± 0.07	1.93 ± 0.07	1.92 ± 0.07	1.90 ± 0.05	1.86 ± 0.05		1.49 ± 0.04	1.43 ± 0.04	1.46 ± 0.06	1.47 ± 0.04	1.46 ± 0.05	1.46 ± 0.06	1.43 ± 0.03	1.42 ± 0.05			
Q3	2.18 ± 0.08	2.14 ± 0.07	2.16 ± 0.06	2.16 ± 0.07	2.13 ± 0.06	2.15 ± 0.06	2.07 ± 0.00	2.11 ± 0.05		1.65 ± 0.06	1.65 ± 0.08	1.65 ± 0.06	1.67 ± 0.05	1.69 ± 0.06	1.66 ± 0.06	1.67 ± 0.06	1.57 ± −			
Q4	2.54 ± 0.24	2.54 ± 0.22	2.49 ± 0.16	2.50 ± 0.18	2.47 ± 0.13	2.43 ± 0.11	2.45 ± 0.04	− ± −		1.99 ± 0.20	1.99 ± 0.19	2.00 ± 0.16	1.90 ± 0.08	1.99 ± 0.13	1.94 ± 0.14	1.86 ± −	− ± −			
Junior																				
Q1	1.81 ± 0.13	1.85 ± 0.16	1.86 ± 0.12	1.82 ± 0.17	1.81 ± 0.17	1.81 ± 0.14	1.79 ± 0.18	1.63 ± 0.27		1.29 ± 0.19	1.35 ± 0.09	1.29 ± 0.16	1.33 ± 0.15	1.29 ± 0.14	1.30 ± 0.11	1.30 ± 0.18	1.24 ± 0.19			
Q2	2.09 ± 0.06	2.14 ± 0.04	2.15 ± 0.06	2.14 ± 0.06	2.12 ± 0.06	2.12 ± 0.06	2.11 ± 0.05	2.10 ± 0.04		1.56 ± 0.04	1.62 ± 0.05	1.61 ± 0.05	1.59 ± 0.06	1.58 ± 0.06	1.58 ± 0.05	1.58 ± 0.07	1.56 ± 0.08			
Q3	2.40 ± 0.05	2.36 ± 0.07	2.35 ± 0.06	2.35 ± 0.06	2.36 ± 0.07	2.34 ± 0.06	2.35 ± 0.05	2.31 ± 0.03		1.81 ± 0.05	1.81 ± 0.05	1.80 ± 0.05	1.79 ± 0.05	1.79 ± 0.06	1.77 ± 0.05	1.77 ± 0.05	1.71 ± −			
Q4	2.67 ± 0.19	2.74 ± 0.17	2.70 ± 0.18	2.68 ± 0.17	2.64 ± 0.15	2.59 ± 0.14	2.60 ± 0.11	− ± −		2.16 ± 0.17	2.14 ± 0.18	2.10 ± 0.16	2.09 ± 0.19	2.09 ± 0.17	2.02 ± 0.14	2.04 ± 0.12	− ± −			
Open																				
Q1	1.67 ± 0.49	1.76 ± 0.17	1.76 ± 0.15	1.82 ± 0.10	1.82 ± 0.15	1.78 ± 0.17	1.71 ± 0.25	1.69 ± 0.24		1.23 ± 0.22	1.35 ± 0.09	1.38 ± 0.06	1.27 ± 0.17	1.30 ± 0.10	1.30 ± 0.14	1.27 ± 0.14	1.15 ± 0.19			
Q2	2.04 ± 0.05	2.15 ± 0.04	2.10 ± 0.06	2.12 ± 0.06	2.10 ± 0.06	2.11 ± 0.07	2.08 ± 0.06	2.09 ± 0.07		1.50 ± 0.03	1.57 ± 0.05	1.58 ± 0.06	1.57 ± 0.06	1.57 ± 0.06	1.56 ± 0.06	1.55 ± 0.05	1.54 ± 0.07			
Q3	2.36 ± 0.06	2.33 ± 0.07	2.35 ± 0.08	2.34 ± 0.07	2.33 ± 0.06	2.33 ± 0.07	2.30 ± 0.06	2.29 ± 0.08		1.82 ± 0.07	1.80 ± 0.06	1.80 ± 0.07	1.77 ± 0.07	1.80 ± 0.07	1.81 ± 0.06	1.79 ± 0.06	1.75 ± 0.05			
Q4	2.95 ± 0.40	2.76 ± 0.19	2.76 ± 0.22	2.69 ± 0.16	2.71 ± 0.17	2.60 ± 0.11	2.61 ± 0.12	− ± −		2.18 ± 0.17	2.15 ± 0.20	2.18 ± 0.19	2.13 ± 0.20	2.08 ± 0.12	2.12 ± 0.16	2.13 ± 0.13	2.08 ± 0.17			
Master 1																				
Q1	1.73 ± −	1.55 ± −	1.58 ± 0.20	1.58 ± 0.10	1.44 ± 0.39	1.52 ± 0.25	1.54 ± 0.12	1.42 ± 0.25		1.02 ± −	1.18 ± 0.05	1.05 ± 0.20	1.13 ± 0.11	1.08 ± 0.11	1.10 ± 0.17	1.10 ± 0.10	1.03 ± 0.15			
Q2	1.93 ± 0.09	1.94 ± 0.10	1.89 ± 0.07	1.90 ± 0.07	1.91 ± 0.07	1.87 ± 0.07	1.89 ± 0.07	1.87 ± 0.07		1.35 ± 0.07	1.35 ± 0.11	1.39 ± 0.06	1.39 ± 0.06	1.38 ± 0.06	1.38 ± 0.04	1.34 ± 0.05	1.35 ± 0.06			
Q3	− ± −	2.18 ± 0.08	2.16 ± 0.08	2.12 ± 0.07	2.15 ± 0.08	2.15 ± 0.06	2.12 ± 0.06	2.11 ± 0.08		1.48 ± −	1.57 ± 0.06	1.61 ± 0.08	1.60 ± 0.07	1.57 ± 0.06	1.59 ± 0.07	1.56 ± 0.05	1.56 ± 0.07			
Q4	2.48 ± 0.15	2.50 ± 0.14	2.52 ± 0.21	2.48 ± 0.17	2.49 ± 0.20	2.50 ± 0.14	2.39 ± 0.07	− ± −		2.04 ± 0.22	2.03 ± 0.34	1.91 ± 0.13	1.90 ± 0.18	1.88 ± 0.10	1.85 ± 0.13	2.01 ± 0.23	1.73 ± 0.04			
Master 2																				
Q1	1.45 ± 0.14	1.55 ± 0.02	1.47 ± 0.15	1.50 ± 0.14	1.47 ± 0.25	1.39 ± 0.19	1.46 ± 0.13	1.43 ± 0.11		− ± −	− ± −	0.93 ± 0.03	0.95 ± 0.16	0.89 ± 0.25	0.77 ± 0.05	0.91 ± 0.12	0.83 ± 0.22			
Q2	1.77 ± 0.07	1.83 ± 0.05	1.81 ± 0.06	1.83 ± 0.04	1.77 ± 0.05	1.83 ± 0.06	1.79 ± 0.07	1.75 ± 0.04		− ± −	1.19 ± 0.11	1.23 ± 0.09	1.22 ± 0.07	1.17 ± 0.07	1.22 ± 0.07	1.20 ± 0.07	1.16 ± 0.06			
Q3	− ± −	2.08 ± 0.07	1.99 ± 0.06	2.05 ± 0.07	2.03 ± 0.07	2.04 ± 0.07	2.01 ± 0.06	1.99 ± 0.07		1.53 ± −	1.46 ± 0.12	1.41 ± 0.08	1.45 ± 0.08	1.51 ± 0.08	1.45 ± 0.06	1.45 ± 0.07	1.45 ± −			
Q4	2.79 ± −	2.48 ± 0.26	2.39 ± 0.17	2.39 ± 0.23	2.36 ± 0.17	2.28 ± 0.08	2.32 ± 0.19	− ± −		1.71 ± 0.16	1.78 ± 0.19	1.85 ± 0.16	1.78 ± 0.09	1.63 ± −	1.81 ± 0.13	1.99 ± −	− ± −			
Master 3																				
Q1	1.17 ± 0.29	0.97 ± 0.26	1.38 ± 0.09	1.29 ± 0.07	1.26 ± 0.37	1.06 ± 0.32	1.00 ± 0.26	1.11 ± 0.30		0.64 ± −	− ± −	0.63 ± −	0.92 ± −	− ± −	0.74 ± 0.05	− ± −	0.89 ± 0.06			
Q2	1.66 ± −	1.65 ± 0.09	1.62 ± 0.05	1.65 ± 0.05	1.62 ± 0.09	1.60 ± 0.09	1.59 ± 0.13	1.63 ± −		− ± −	− ± −	1.07 ± −	1.07 ± 0.05	0.98 ± 0.05	1.14 ± −	− ± −	− ± −			
Q3	1.90 ± −	1.87 ± 0.12	1.89 ± 0.06	1.89 ± 0.07	1.90 ± 0.07	1.96 ± 0.03	1.92 ± 0.07	− ± −		− ± −	− ± −	1.34 ± 0.01	1.31 ± 0.01	1.27 ± 0.09	1.18 ± −	− ± −	1.28 ± −			
Q4	2.07 ± −	2.18 ± 0.04	2.35 ± 0.29	2.24 ± 0.22	2.24 ± 0.10	2.06 ± 0.03	− ± −	− ± −		− ± −	2.11 ± −	1.42 ± 0.03	1.60 ± −	1.58 ± 0.24	1.43 ± 0	1.58 ± 0.02	− ± −			
Master 4																				
Q1	− ± −	0.92 ± −	0.91 ± 0.16	0.75 ± 0.23	1.07 ± 0.12	0.92 ± 0.17	− ± −	1.00 ± −		− ± −	− ± −	− ± −	0.48 ± −	− ± −	− ± −	0.97 ± −	− ± −			
Q2	1.40 ± 0.11	− ± −	1.35 ± 0.12	1.35 ± 0.10	1.25 ± 0.07	1.34 ± 0	1.32 ± 0.15	− ± −		− ± −	− ± −	1.39 ± −	1.08 ± −	− ± −	− ± −	− ± −	− ± −			
Q3	1.69 ± −	1.54 ± 0.03	1.67 ± 0.00	1.64 ± 0.06	1.52 ± −	− ± −	− ± −	− ± −		− ± −	1.4 ± −	− ± −	1.39 ± −	− ± −	− ± −	− ± −	− ± −			
Q4	− ± −	2.04 ± 0.16	1.85 ± 0.12	2.02 ± 0.08	− ± −	1.81 ± −	− ± −	− ± −		− ± −	1.44 ± −	− ± −	− ± −	− ± −	− ± −	− ± −	− ± −			

Q1 identified the weakest athletes in the bottom 25th percentile, Q2 and Q3 identified athletes within the 25 and 50th and the 50 to 75th percentile, respectively, while Q4 identified the strongest athletes in the > 75th percentile. Sub-junior identifies athletes ≤ 18 years, Junior > 18 ≤ 23 years, Open > 23 ≤ 39 years, Masters 1 > 39 ≤ 49 years, Masters 2 > 49 ≤ 59 years, Masters 3 > 59 ≤ 69 years, and Masters 4 > 69 years. Females and Males athletes are divided in weight categories and expressed in kilograms (Females: 47 kg, 52 kg, 57 kg, 63 kg, 69 kg, 76 kg, 84 kg, and > 84 kg; males: 59 kg, 66 kg, 74 kg, 83 kg, 93 kg, 105 kg, 120 kg, and > 120 kg). “− ± −“ indicates a lack of data to compute the mean ± Standard deviation

**Table 1:** **B**: bench press normative data for European powerlifting athletes divided by sex, age, weight categories, and quartiles. Data are presented as mean ± standard deviation

	Males									Females										
	Bench Press																	Main Effect		
	59 kg	66 kg	74 kg	83 kg	93 kg	105 kg	-120 kg	120 kg		47 kg	52 kg	57 kg	63 kg	69 kg	76 kg	-84 kg	84 kg	Sex	Age cat.	Quartile
Sub-junior																		< 0.001	< 0.001	< 0.001
Q1	0.97 ± 0.16	1.02 ± 0.12	1.05 ± 0.09	1.04 ± 0.10	1.04 ± 0.09	1.02 ± 0.12	0.95 ± 0.13	0.84 ± 0.17		0.65 ± 0.05	0.64 ± 0.04	0.63 ± 0.08	0.64 ± 0.09	0.63 ± 0.06	0.63 ± 0.07	0.66 ± 0.06	0.58 ± 0.10			
Q2	1.25 ± 0.04	1.25 ± 0.04	1.24 ± 0.04	1.24 ± 0.04	1.24 ± 0.04	1.24 ± 0.04	1.24 ± 0.04	1.24 ± 0.06		0.78 ± 0.05	0.81 ± 0.03	0.79 ± 0.03	0.80 ± 0.04	0.78 ± 0.03	0.81 ± 0.03	0.79 ± 0.02	0.78 ± 0.04			
Q3	1.39 ± 0.04	1.39 ± 0.04	1.39 ± 0.04	1.39 ± 0.04	1.39 ± 0.04	1.41 ± 0.04	1.37 ± 0.05	1.35 ± 0.01		0.94 ± 0.03	0.93 ± 0.04	0.93 ± 0.03	0.92 ± 0.04	0.93 ± 0.04	0.91 ± 0.03	0.91 ± 0.04	0.93 ± −			
Q4	1.66 ± 0.15	1.64 ± 0.20	1.64 ± 0.14	1.62 ± 0.13	1.63 ± 0.14	1.55 ± 0.05	1.59 ± 0.13	− ± −		1.08 ± 0.08	1.13 ± 0.10	1.13 ± 0.09	1.11 ± 0.11	1.12 ± 0.12	1.12 ± 0.11	1.10 ± −	− ± −			
Junior																				
Q1	1.17 ± 0.11	1.18 ± 0.08	1.21 ± 0.07	1.16 ± 0.11	1.16 ± 0.12	1.16 ± 0.11	1.11 ± 0.14	0.99 ± 0.16		0.71 ± 0.10	0.75 ± 0.05	0.72 ± 0.05	0.69 ± 0.07	0.70 ± 0.05	0.70 ± 0.06	0.67 ± 0.07	0.63 ± 0.08			
Q2	1.37 ± 0.04	1.39 ± 0.05	1.39 ± 0.04	1.38 ± 0.04	1.39 ± 0.04	1.39 ± 0.04	1.37 ± 0.04	1.38 ± 0.05		0.87 ± 0.04	0.86 ± 0.04	0.85 ± 0.03	0.86 ± 0.03	0.85 ± 0.04	0.86 ± 0.03	0.85 ± 0.02	0.84 ± 0.04			
Q3	1.56 ± 0.05	1.55 ± 0.04	1.55 ± 0.05	1.54 ± 0.05	1.54 ± 0.05	1.55 ± 0.05	1.52 ± 0.05	1.51 ± 0.03		1.00 ± 0.04	0.99 ± 0.04	0.99 ± 0.04	0.98 ± 0.03	0.98 ± 0.03	0.96 ± 0.04	0.98 ± 0.04	0.97 ± −			
Q4	1.82 ± 0.14	1.83 ± 0.15	1.81 ± 0.17	1.81 ± 0.13	1.77 ± 0.13	1.76 ± 0.12	1.78 ± 0.11	− ± −		1.25 ± 0.13	1.23 ± 0.15	1.20 ± 0.10	1.22 ± 0.16	1.22 ± 0.12	1.22 ± 0.12	1.16 ± 0.08	1.14 ± −			
Open																				
Q1	1.19 ± 0.21	1.21 ± 0.14	1.21 ± 0.09	1.19 ± 0.11	1.20 ± 0.09	1.17 ± 0.13	1.12 ± 0.16	1.15 ± 0.13		0.65 ± −	0.71 ± 0.05	0.72 ± 0.04	0.70 ± 0.05	0.70 ± 0.06	0.70 ± 0.07	0.70 ± 0.07	0.63 ± 0.08			
Q2	1.45 ± 0.04	1.43 ± 0.05	1.42 ± 0.06	1.42 ± 0.05	1.42 ± 0.05	1.43 ± 0.05	1.42 ± 0.05	1.41 ± 0.05		0.85 ± 0.02	0.88 ± 0.03	0.87 ± 0.04	0.87 ± 0.04	0.87 ± 0.04	0.86 ± 0.04	0.87 ± 0.04	0.86 ± 0.04			
Q3	1.58 ± 0.05	1.61 ± 0.06	1.61 ± 0.05	1.62 ± 0.05	1.61 ± 0.05	1.61 ± 0.05	1.62 ± 0.05	1.58 ± 0.05		1.03 ± 0.05	1.01 ± 0.04	1.02 ± 0.04	1.01 ± 0.04	1.01 ± 0.04	1.02 ± 0.04	1.02 ± 0.05	1.00 ± 0.05			
Q4	2.08 ± 0.22	1.92 ± 0.14	1.91 ± 0.16	1.87 ± 0.13	1.88 ± 0.13	1.84 ± 0.10	1.86 ± 0.12	1.91 ± 0.08		1.36 ± 0.19	1.31 ± 0.15	1.28 ± 0.15	1.25 ± 0.13	1.22 ± 0.09	1.26 ± 0.13	1.18 ± 0.05	1.19 ± 0.11			
Master 1																				
Q1	1.13 ± −	− ± −	1.19 ± 0.09	1.09 ± 0.17	1.11 ± 0.16	1.09 ± 0.12	1.14 ± 0.11	1.04 ± 0.21		0.61 ± 0.10	0.66 ± 0.02	0.69 ± 0.03	0.66 ± 0.06	0.67 ± 0.03	0.62 ± 0.04	0.64 ± 0.04	0.59 ± 0.09			
Q2	− ± −	1.34 ± 0.06	1.38 ± 0.04	1.37 ± 0.05	1.37 ± 0.06	1.38 ± 0.04	1.38 ± 0.04	1.36 ± 0.05		− ± −	0.79 ± 0.05	0.81 ± 0.02	0.81 ± 0.04	0.76 ± 0.05	0.80 ± 0.04	0.78 ± 0.03	0.76 ± 0.03			
Q3	1.51 ± 0.07	1.54 ± 0.05	1.55 ± 0.05	1.55 ± 0.04	1.54 ± 0.05	1.54 ± 0.05	1.53 ± 0.04	1.51 ± 0.05		0.86 ± −	0.95 ± 0.06	0.95 ± 0.03	0.93 ± 0.03	0.93 ± 0.04	0.93 ± 0.03	0.95 ± 0.04	0.92 ± 0.05			
Q4	1.91 ± 0.19	1.84 ± 0.18	1.85 ± 0.16	1.78 ± 0.11	1.85 ± 0.16	1.77 ± 0.10	1.71 ± 0.04	1.72 ± −		1.19 ± 0.15	1.23 ± 0.18	1.18 ± 0.18	1.18 ± 0.11	1.09 ± 0.05	1.18 ± 0.17	1.25 ± 0.24	1.14 ± 0.13			
Master 2																				
Q1	1.12 ± 0.06	1.03 ± −	1.10 ± 0.06	1.15 ± 0.05	1.10 ± 0.07	1.04 ± 0.17	1.05 ± 0.11	0.96 ± 0.12		− ± −	− ± −	0.65 ± −	0.63 ± 0.05	0.60 ± 0.04	0.64 ± 0.02	0.55 ± 0.08	0.57 ± 0.06			
Q2	− ± −	1.29 ± 0.04	1.29 ± 0.04	1.30 ± 0.05	1.27 ± 0.04	1.30 ± 0.04	1.30 ± 0.04	1.29 ± 0.04		− ± −	0.77 ± −	0.73 ± 0.03	0.72 ± 0.04	0.70 ± 0.02	0.73 ± 0.04	0.71 ± 0.02	0.71 ± 0.04			
Q3	1.50 ± −	1.46 ± 0.06	1.43 ± 0.05	1.46 ± 0.05	1.45 ± 0.04	1.47 ± 0.05	1.48 ± 0.04	1.48 ± 0.08		0.87 ± −	0.95 ± 0.02	0.89 ± 0.06	0.90 ± 0.06	0.86 ± −	0.91 ± 0.04	0.89 ± 0.06	0.91 ± −			
Q4	1.63 ± 0.08	1.70 ± 0.17	1.75 ± 0.14	1.72 ± 0.13	1.68 ± 0.11	1.63 ± 0.10	1.70 ± 0.18	1.55 ± −		1.11 ± 0.01	1.23 ± 0.11	1.12 ± 0.16	1.14 ± 0.09	1.07 ± 0.11	1.12 ± 0.12	1.09 ± −	1.05 ± −			
Master 3																				
Q1	0.80 ± 0.13	1.02 ± 0.05	0.96 ± 0	0.98 ± 0.10	0.91 ± 0.11	0.98 ± 0.04	0.90 ± 0.12	0.96 ± 0.03		0.58 ± −	− ± −	− ± −	0.53 ± 0.04	0.50 ± 0.07	0.46 ± 0.00	− ± −	0.55 ± −			
Q2	1.21 ± −	1.21 ± −	1.18 ± 0.05	1.17 ± 0.06	1.16 ± 0.03	1.12 ± 0.02	1.25 ± −	1.25 ± 0.00		− ± −	− ± −	0.67 ± 0.06	0.65 ± 0.07	0.64 ± −	0.68 ± 0.06	− ± −	0.64 ± −			
Q3	1.38 ± −	1.34 ± 0.02	1.33 ± 0.05	1.34 ± 0.04	1.33 ± 0.04	1.32 ± 0.04	1.35 ± 0.07	1.28 ± −		− ± −	− ± −	0.79 ± 0.02	0.76 ± 0.01	0.76 ± 0.02	0.78 ± −	0.77 ± −	0.81 ± −			
Q4	− ± −	1.60 ± 0.10	1.51 ± 0.11	1.58 ± 0.14	1.60 ± 0.11	1.57 ± 0.13	1.46 ± −	1.45 ± −		− ± −	1.05 ± −	0.89 ± 0.04	0.83 ± 0.00	0.85 ± −	0.94 ± −	1.21 ± −	− ± −			
Master 4																				
Q1	− ± −	0.73 ± −	0.89 ± 0.00	0.78 ± 0.10	0.87 ± −	− ± −	0.84 ± −	0.83 ± −		− ± −	− ± −	− ± −	0.52 ± −	− ± −	− ± −	0.62 ± −	− ± −			
Q2	− ± −	1.00 ± 0.02	0.97 ± 0.07	0.94 ± 0.04	0.98 ± 0.02	0.95 ± 0.05	− ± −	− ± −		− ± −	0.9 ± −	0.69 ± −	− ± −	− ± −	− ± −	− ± −	− ± −			
Q3	1.05 ± −	1.16 ± 0.01	1.12 ± 0.05	1.14 ± 0.04	1.08 ± −	1.09 ± −	1.07 ± −	− ± −		− ± −	0.92 ± −	− ± −	0.90 ± −	− ± −	− ± −	− ± −	− ± −			
Q4	1.20 ± 0.03	1.23 ± 0.03	1.52 ± 0.01	1.25 ± 0.06	− ± −	1.23 ± −	− ± −	− ± −		− ± −	− ± −	− ± −	1.00 ± −	− ± −	− ± −	− ± −	− ± −			

Q1 identified the weakest athletes in the bottom 25th percentile, Q2 and Q3 identified athletes within the 25 and 50th and the 50 to 75th percentile, respectively, while Q4 identified the strongest athletes in the > 75th percentile. Sub-junior identifies athletes ≤ 18 years, Junior > 18 ≤ 23 years, Open > 23 ≤ 39 years, Masters 1 > 39 ≤ 49 years, Masters 2 > 49 ≤ 59 years, Masters 3 > 59 ≤ 69 years, and Masters 4 > 69 years. Females and Males athletes are divided in weight categories and expressed in kilograms (Females: 47 kg, 52 kg, 57 kg, 63 kg, 69 kg, 76 kg, 84 kg, and > 84 kg; males: 59 kg, 66 kg, 74 kg, 83 kg, 93 kg, 105 kg, 120 kg, and > 120 kg). “− ± −“ indicates a lack of data to compute the mean ± Standard deviation

**Table 1:** **C**: deadlift normative data for European powerlifting athletes divided by sex, age, weight categories, and quartiles. Data are presented as mean ± standard deviation

	Males									Females										
	Deadlift																	Main Effect		
	59 kg	66 kg	74 kg	83 kg	93 kg	105 kg	-120 kg	120 kg		47 kg	52 kg	57 kg	63 kg	69 kg	76 kg	-84 kg	84 kg	Sex	Age cat.	Quartile
Sub-junior																		< 0.001	< 0.001	< 0.001
Q1	1.90 ± 0.15	1.87 ± 0.21	1.88 ± 0.26	1.95 ± 0.25	1.96 ± 0.20	1.95 ± 0.21	1.81 ± 0.21	1.63 ± 0.36		1.33 ± 0.11	1.54 ± 0.07	1.47 ± 0.18	1.44 ± 0.19	1.52 ± 0.09	1.47 ± 0.18	1.46 ± 0.16	1.28 ± 0.20			
Q2	2.31 ± 0.08	2.29 ± 0.07	2.34 ± 0.07	2.32 ± 0.07	2.32 ± 0.07	2.31 ± 0.07	2.32 ± 0.08	2.31 ± 0.02		1.77 ± 0.07	1.80 ± 0.08	1.80 ± 0.06	1.77 ± 0.07	1.79 ± 0.08	1.76 ± 0.08	1.78 ± 0.04	1.76 ± 0.10			
Q3	2.60 ± 0.07	2.59 ± 0.08	2.60 ± 0.07	2.58 ± 0.07	2.56 ± 0.07	2.58 ± 0.07	2.56 ± 0.03	− ± −		2.08 ± 0.06	2.01 ± 0.06	2.06 ± 0.05	2.01 ± 0.06	2.06 ± 0.04	2.01 ± 0.06	1.96 ± 0.01	2.02 ± −			
Q4	3.04 ± 0.20	2.99 ± 0.21	3.00 ± 0.22	2.94 ± 0.16	2.89 ± 0.14	2.89 ± 0.04	2.74 ± 0.01	− ± −		2.35 ± 0.12	2.43 ± 0.13	2.37 ± 0.13	2.35 ± 0.11	2.25 ± 0.06	2.40 ± 0.17	2.19 ± 0.04	− ± −			
Junior																				
Q1	2.20 ± 0.23	2.17 ± 0.12	2.23 ± 0.12	2.17 ± 0.20	2.17 ± 0.23	2.17 ± 0.19	2.10 ± 0.20	1.88 ± 0.30		1.47 ± 0.26	1.60 ± 0.15	1.62 ± 0.13	1.65 ± 0.14	1.61 ± 0.14	1.63 ± 0.13	1.61 ± 0.17	1.45 ± 0.17			
Q2	2.52 ± 0.08	2.55 ± 0.05	2.52 ± 0.07	2.51 ± 0.06	2.52 ± 0.06	2.52 ± 0.06	2.51 ± 0.07	2.41 ± −		1.88 ± −	1.95 ± 0.06	1.95 ± 0.06	1.93 ± 0.06	1.91 ± 0.06	1.93 ± 0.06	1.91 ± 0.05	1.87 ± 0.03			
Q3	2.77 ± 0.08	2.79 ± 0.05	2.78 ± 0.07	2.76 ± 0.07	2.76 ± 0.06	2.73 ± 0.06	2.76 ± 0.07	− ± −		2.12 ± 0.05	2.15 ± 0.07	2.19 ± 0.07	2.18 ± 0.06	2.17 ± 0.07	2.15 ± 0.07	2.12 ± 0.05	− ± −			
Q4	3.29 ± 0.24	3.23 ± 0.26	3.15 ± 0.19	3.12 ± 0.21	3.08 ± 0.15	3.05 ± 0.11	− ± −	− ± −		2.70 ± 0.25	2.58 ± 0.25	2.54 ± 0.15	2.49 ± 0.17	2.51 ± 0.18	2.45 ± 0.12	2.48 ± 0.21	− ± −			
Open																				
Q1	0.77 ± −	2.23 ± 0.08	2.04 ± 0.23	2.20 ± 0.11	2.15 ± 0.15	2.12 ± 0.18	2.08 ± 0.20	1.92 ± 0.28		− ± −	1.66 ± 0.11	1.77 ± 0.02	1.63 ± 0.18	1.64 ± 0.12	1.61 ± 0.14	1.61 ± 0.13	1.41 ± 0.21			
Q2	2.39 ± 0.05	2.49 ± 0.07	2.49 ± 0.07	2.49 ± 0.07	2.48 ± 0.08	2.47 ± 0.07	2.46 ± 0.07	2.43 ± 0.07		1.93 ± 0.07	1.98 ± 0.06	1.93 ± 0.08	1.96 ± 0.07	1.93 ± 0.07	1.93 ± 0.08	1.90 ± 0.08	1.92 ± 0.08			
Q3	2.79 ± 0.07	2.76 ± 0.08	2.76 ± 0.08	2.76 ± 0.07	2.75 ± 0.08	2.74 ± 0.08	2.71 ± 0.09	2.71 ± −		2.20 ± 0.06	2.22 ± 0.08	2.24 ± 0.07	2.21 ± 0.08	2.20 ± 0.08	2.18 ± 0.07	2.20 ± 0.07	2.24 ± 0.11			
Q4	3.30 ± 0.24	3.24 ± 0.23	3.26 ± 0.29	3.18 ± 0.22	3.10 ± 0.17	3.03 ± 0.09	3.13 ± −	− ± −		2.67 ± 0.18	2.69 ± 0.24	2.59 ± 0.20	2.56 ± 0.17	2.51 ± 0.11	2.53 ± 0.15	2.45 ± 0.09	2.66 ± 0.24			
Master 1																				
Q1	− ± −	− ± −	1.96 ± 0.12	1.83 ± 0.16	1.67 ± 0.47	1.89 ± 0.18	1.83 ± 0.18	1.70 ± 0.25		1.51 ± −	− ± −	1.56 ± 0.10	1.50 ± 0.16	1.58 ± 0.04	1.47 ± 0.13	1.47 ± 0.14	1.38 ± 0.15			
Q2	− ± −	2.27 ± 0.12	2.22 ± 0.07	2.25 ± 0.10	2.27 ± 0.09	2.24 ± 0.09	2.21 ± 0.10	2.19 ± 0.09		− ± −	1.77 ± 0.06	1.72 ± 0.05	1.83 ± 0.06	1.77 ± 0.07	1.80 ± 0.08	1.77 ± 0.07	1.76 ± 0.07			
Q3	2.50 ± 0.14	2.55 ± 0.10	2.57 ± 0.09	2.55 ± 0.09	2.54 ± 0.08	2.52 ± 0.07	2.52 ± 0.07	− ± −		1.91 ± −	2.01 ± 0.07	2.07 ± 0.06	2.04 ± 0.07	2.06 ± 0.06	2.02 ± 0.07	2.06 ± 0.05	1.97 ± 0.07			
Q4	3.14 ± 0.28	3.05 ± 0.19	3.05 ± 0.30	2.98 ± 0.16	2.92 ± 0.18	2.87 ± 0.12	2.71 ± −	− ± −		2.59 ± 0.26	2.53 ± 0.34	2.41 ± 0.24	2.37 ± 0.15	2.27 ± 0.11	2.34 ± 0.13	2.30 ± 0.12	− ± −			
Master 2																				
Q1	1.86 ± −	1.80 ± 0.16	1.78 ± 0.16	1.71 ± 0.23	1.75 ± 0.21	1.73 ± 0.26	1.73 ± 0.20	1.73 ± 0.14		− ± −	− ± −	1.40 ± 0.14	1.34 ± 0.10	1.45 ± 0.05	1.33 ± −	1.29 ± 0.13	1.24 ± 0.16			
Q2	2.18 ± 0.12	2.23 ± 0.01	2.17 ± 0.05	2.16 ± 0.09	2.15 ± 0.07	2.13 ± 0.06	2.10 ± 0.06	2.11 ± 0.09		− ± −	1.72 ± −	1.69 ± 0.07	1.60 ± 0.09	1.64 ± 0.09	1.62 ± 0.09	1.67 ± 0.04	1.59 ± 0.08			
Q3	− ± −	2.48 ± 0.08	2.40 ± 0.07	2.40 ± 0.09	2.43 ± 0.09	2.38 ± 0.08	2.39 ± 0.08	− ± −		2.05 ± 0.12	− ± −	2.02 ± 0.12	1.91 ± 0.11	1.93 ± 0.07	1.95 ± 0.10	− ± −	1.78 ± −			
Q4	2.72 ± 0.22	2.90 ± 0.32	2.85 ± 0.24	2.80 ± 0.18	2.75 ± 0.13	2.69 ± 0.08	2.69 ± −	− ± −		2.58 ± −	2.35 ± 0.17	2.41 ± 0.15	2.27 ± 0.11	2.23 ± 0.02	2.29 ± 0.10	2.61 ± −	− ± −			
Master 3																				
Q1	1.60 ± 0.17	1.55 ± 0.05	1.91 ± −	1.76 ± 0.20	1.68 ± 0.30	1.48 ± 0.42	1.56 ± 0.38	1.60 ± 0.12		1.28 ± −	− ± −	1.44 ± −	1.44 ± −	− ± −	1.38 ± 0.06	− ± −	1.23 ± 0.11			
Q2	− ± −	2.14 ± −	2.09 ± 0.04	2.13 ± 0.07	2.06 ± 0.08	2.02 ± 0.07	2.01 ± 0.01	− ± −		− ± −	− ± −	1.69 ± −	1.64 ± 0.05	1.61 ± −	1.57 ± 0	− ± −	1.58 ± −			
Q3	2.24 ± −	2.37 ± 0.09	2.40 ± 0.08	2.30 ± 0.05	2.37 ± 0.07	2.37 ± 0.07	2.32 ± −	− ± −		− ± −	− ± −	1.88 ± 0.04	1.80 ± 0.10	1.72 ± −	1.72 ± −	1.84 ± 0.01	− ± −			
Q4	2.75 ± 0.09	2.71 ± 0.18	2.78 ± 0.31	2.72 ± 0.17	2.71 ± 0.23	− ± −	− ± −	− ± −		− ± −	2.71 ± −	2.13 ± 0.18	− ± −	2.10 ± 0.17	2.09 ± −	− ± −	− ± −			
Master 4																				
Q1	1.67 ± −	− ± −	1.57 ± −	1.28 ± 0.46	1.60 ± 0.06	1.50 ± −	1.65 ± −	1.24 ± −		− ± −	− ± −	− ± −	0.80 ± −	− ± −	− ± −	1.19 ± −	− ± −			
Q2	− ± −	1.86 ± 0.02	1.71 ± −	1.84 ± 0.09	1.84 ± 0.03	1.84 ± 0.11	1.87 ± −	− ± −		− ± −	− ± −	2.03 ± −	1.83 ± −	− ± −	− ± −	− ± −	− ± −			
Q3	− ± −	2.17 ± 0.09	2.28 ± 0.06	2.26 ± 0.06	− ± −	− ± −	− ± −	− ± −		− ± −	2.2 ± −	− ± −	2.17 ± −	− ± −	− ± −	− ± −	− ± −			
Q4	2.43 ± 0.08	2.71 ± 0.18	2.46 ± 0.05	2.88 ± 0.39	− ± −	− ± −	− ± −	− ± −		− ± −	2.21 ± −	− ± −	− ± −	− ± −	− ± −	− ± −	− ± −			

Q1 identified the weakest athletes in the bottom 25th percentile, Q2 and Q3 identified athletes within the 25 and 50th and the 50 to 75th percentile, respectively, while Q4 identified the strongest athletes in the > 75th percentile. Sub-junior identifies athletes ≤ 18 years, Junior > 18 ≤ 23 years, Open > 23 ≤ 39 years, Masters 1 > 39 ≤ 49 years, Masters 2 > 49 ≤ 59 years, Masters 3 > 59 ≤ 69 years, and Masters 4 > 69 years. Females and Males athletes are divided in weight categories and expressed in kilograms (Females: 47 kg, 52 kg, 57 kg, 63 kg, 69 kg, 76 kg, 84 kg, and > 84 kg; males: 59 kg, 66 kg, 74 kg, 83 kg, 93 kg, 105 kg, 120 kg, and > 120 kg). “− ± −“ indicates a lack of data to compute the mean ± Standard deviation

**Table 1:** **D**: total normative data for European powerlifting athletes divided by sex, age, weight categories, and quartiles. Data are presented as mean ± standard deviation

	Males									Females										
	Total																	Main Effect		
	59 kg	66 kg	74 kg	83 kg	93 kg	105 kg	-120 kg	120 kg		47 kg	52 kg	57 kg	63 kg	69 kg	76 kg	-84 kg	84 kg	Sex	Age cat.	Quartile
Sub-junior																		< 0.001	< 0.001	< 0.001
Q1	4.42 ± 0.45	4.66 ± 0.45	4.67 ± 0.52	4.66 ± 0.45	4.70 ± 0.46	4.67 ± 0.49	4.45 ± 0.53	3.91 ± 0.78		3.19 ± 0.22	3.48 ± 0.21	3.35 ± 0.40	3.44 ± 0.36	3.51 ± 0.19	3.33 ± 0.37	3.32 ± 0.33	3.02 ± 0.39			
Q2	5.55 ± 0.18	5.54 ± 0.18	5.55 ± 0.16	5.55 ± 0.17	5.52 ± 0.17	5.48 ± 0.16	5.31 ± 0.05	5.51 ± 0.06		4.19 ± 0.06	4.09 ± 0.17	4.12 ± 0.15	4.07 ± 0.18	4.11 ± 0.19	4.03 ± 0.14	4.01 ± 0.20	4.00 ± 0.12			
Q3	6.13 ± 0.18	6.13 ± 0.19	6.13 ± 0.17	6.10 ± 0.17	6.11 ± 0.17	6.06 ± 0.17	6.09 ± 0.22	− ± −		4.62 ± 0.12	4.64 ± 0.14	4.59 ± 0.15	4.64 ± 0.16	4.59 ± 0.18	4.64 ± 0.15	4.65 ± 0.14	4.36 ± −			
Q4	7.07 ± 0.43	7.08 ± 0.56	7.03 ± 0.42	6.93 ± 0.39	6.81 ± 0.34	6.70 ± 0.24	6.51 ± −	− ± −		5.26 ± 0.43	5.43 ± 0.39	5.41 ± 0.31	5.24 ± 0.20	5.18 ± 0.23	5.36 ± 0.44	− ± −	− ± −			
Junior																				
Q1	5.34 ± 0.29	5.28 ± 0.39	5.43 ± 0.26	5.30 ± 0.43	5.29 ± 0.45	5.26 ± 0.39	5.13 ± 0.48	4.67 ± 0.76		2.84 ± −	3.57 ± 0.40	3.66 ± 0.27	3.81 ± 0.39	3.70 ± 0.33	3.78 ± 0.31	3.67 ± 0.38	3.41 ± 0.44			
Q2	6.09 ± 0.13	6.10 ± 0.19	6.14 ± 0.17	6.10 ± 0.17	6.09 ± 0.16	6.09 ± 0.15	6.02 ± 0.16	5.92 ± 0.15		4.33 ± 0.14	4.49 ± 0.16	4.45 ± 0.16	4.44 ± 0.14	4.42 ± 0.16	4.45 ± 0.13	4.41 ± 0.15	4.24 ± 0.09			
Q3	6.68 ± 0.19	6.64 ± 0.16	6.66 ± 0.17	6.65 ± 0.15	6.63 ± 0.16	6.61 ± 0.15	6.56 ± 0.11	− ± −		5.02 ± 0.09	4.93 ± 0.12	4.95 ± 0.13	4.90 ± 0.14	4.92 ± 0.11	4.91 ± 0.12	4.97 ± 0.13	− ± −			
Q4	7.56 ± 0.51	7.63 ± 0.49	7.53 ± 0.44	7.52 ± 0.43	7.33 ± 0.35	7.40 ± 0.52	7.29 ± 0.26	− ± −		6.07 ± 0.50	5.75 ± 0.54	5.73 ± 0.36	5.69 ± 0.47	5.68 ± 0.44	5.67 ± 0.39	5.59 ± 0.31	− ± −			
Open																				
Q1	4.41 ± 1.15	5.42 ± 0.29	5.23 ± 0.42	5.32 ± 0.29	5.30 ± 0.40	5.17 ± 0.47	5.07 ± 0.56	4.93 ± 0.61		3.90 ± −	3.75 ± 0.19	3.99 ± 0.02	3.69 ± 0.38	3.75 ± 0.25	3.70 ± 0.35	3.66 ± 0.33	3.27 ± 0.47			
Q2	6.16 ± 0.01	6.18 ± 0.14	5.99 ± 0.19	6.07 ± 0.17	6.05 ± 0.18	6.04 ± 0.17	6.02 ± 0.18	6.03 ± 0.17		4.36 ± 0.15	4.50 ± 0.13	4.40 ± 0.18	4.46 ± 0.16	4.45 ± 0.16	4.46 ± 0.16	4.32 ± 0.20	4.29 ± 0.18			
Q3	6.67 ± 0.24	6.81 ± 0.19	6.68 ± 0.18	6.70 ± 0.21	6.68 ± 0.20	6.65 ± 0.17	6.63 ± 0.17	6.43 ± −		5.01 ± 0.15	5.06 ± 0.16	5.05 ± 0.19	5.00 ± 0.19	5.01 ± 0.18	5.04 ± 0.18	4.99 ± 0.14	4.94 ± 0.19			
Q4	8.19 ± 0.83	7.84 ± 0.48	7.78 ± 0.60	7.61 ± 0.43	7.54 ± 0.43	7.38 ± 0.20	7.47 ± 0.17	− ± −		6.11 ± 0.51	6.00 ± 0.49	5.96 ± 0.46	5.86 ± 0.42	5.68 ± 0.29	5.80 ± 0.38	5.62 ± 0.20	6.14 ± −			
Master 1																				
Q1	− ± −	− ± −	5.02 ± 0.20	4.61 ± 0.38	4.47 ± 0.82	4.68 ± 0.50	4.64 ± 0.42	4.34 ± 0.68		3.08 ± −	3.67 ± 0.05	3.39 ± 0.45	3.36 ± 0.18	3.49 ± 0.17	3.23 ± 0.35	3.24 ± 0.26	3.07 ± 0.36			
Q2	5.74 ± −	5.79 ± 0.09	5.55 ± 0.17	5.58 ± 0.23	5.60 ± 0.17	5.55 ± 0.19	5.56 ± 0.17	5.37 ± 0.16		4.09 ± −	4.08 ± 0.19	3.97 ± 0.13	4.10 ± 0.17	3.99 ± 0.20	4.05 ± 0.15	3.99 ± 0.16	3.89 ± 0.15			
Q3	6.29 ± 0.07	6.12 ± 0.17	6.23 ± 0.14	6.19 ± 0.21	6.21 ± 0.18	6.24 ± 0.20	6.14 ± 0.21	5.96 ± 0.12		4.51 ± −	4.64 ± 0.18	4.57 ± 0.19	4.53 ± 0.19	4.57 ± 0.19	4.53 ± 0.17	4.51 ± 0.16	4.46 ± 0.13			
Q4	7.31 ± 0.50	7.23 ± 0.31	7.30 ± 0.60	7.03 ± 0.39	7.23 ± 0.43	6.91 ± 0.28	6.66 ± 0.05	− ± −		5.80 ± 0.53	5.61 ± 0.91	5.38 ± 0.38	5.30 ± 0.37	5.27 ± 0.15	5.26 ± 0.30	5.59 ± 0.58	− ± −			
Master 2																				
Q1	4.92 ± 0.00	4.80 ± −	4.38 ± 0.49	4.71 ± 0.29	4.47 ± 0.40	4.34 ± 0.47	4.44 ± 0.32	4.28 ± 0.35		− ± −	− ± −	2.88 ± −	2.93 ± 0.29	2.93 ± 0.24	3.00 ± 0.19	2.82 ± 0.31	2.75 ± 0.43			
Q2	5.34 ± 0.17	5.33 ± 0.29	5.27 ± 0.24	5.34 ± 0.20	5.23 ± 0.18	5.29 ± 0.19	5.24 ± 0.20	5.26 ± 0.21		− ± −	3.79 ± −	3.65 ± 0.22	3.59 ± 0.28	3.46 ± 0.20	3.60 ± 0.14	3.59 ± 0.19	3.59 ± 0.27			
Q3	− ± −	5.93 ± 0.12	5.86 ± 0.20	5.85 ± 0.18	5.83 ± 0.19	5.90 ± 0.16	5.75 ± 0.16	5.71 ± 0.07		4.38 ± −	4.52 ± 0.06	4.37 ± 0.17	4.27 ± 0.24	4.20 ± 0.15	4.28 ± 0.19	4.03 ± −	− ± −			
Q4	7.17 ± −	7.08 ± 0.74	6.83 ± 0.35	6.69 ± 0.41	6.61 ± 0.35	6.45 ± 0.14	6.60 ± 0.41	− ± −		5.19 ± 0.13	5.47 ± 0.24	5.26 ± 0.32	5.04 ± 0.22	4.90 ± 0.20	5.18 ± 0.30	5.70 ± −	− ± −			
Master 3																				
Q1	3.51 ± 0.40	3.63 ± 0.50	− ± −	4.26 ± 0.26	4.21 ± 0.54	3.87 ± 0.64	3.81 ± 0.57	3.88 ± 0.35		2.50 ± −	− ± −	2.94 ± −	− ± −	3.01 ± −	2.66 ± 0.08	− ± −	2.72 ± 0.25			
Q2	− ± −	4.91 ± 0.24	5.06 ± 0.15	5.07 ± 0.17	4.91 ± 0.24	4.95 ± 0.27	4.95 ± −	4.58 ± −		− ± −	− ± −	3.39 ± −	3.38 ± 0.22	− ± −	3.65 ± −	− ± −	− ± −			
Q3	5.55 ± 0.26	5.60 ± 0.18	5.62 ± 0.09	5.54 ± 0.18	5.53 ± 0.13	5.50 ± 0.07	5.46 ± 0.18	− ± −		− ± −	− ± −	4.00 ± −	4.00 ± 0.14	3.88 ± 0.11	3.74 ± 0	− ± −	3.67 ± −			
Q4	5.84 ± −	6.27 ± 0.25	6.57 ± 0.56	6.14 ± 0.29	6.24 ± 0.33	5.87 ± 0.09	− ± −	− ± −		− ± −	5.88 ± −	4.30 ± 0.15	− ± −	4.94 ± −	4.23 ± −	4.42 ± 0.27	− ± −			
Master 4																				
Q1	− ± −	− ± −	3.79 ± 0.11	3.22 ± 0.54	3.64 ± 0.17	3.52 ± 0.16	− ± −	3.08 ± −		− ± −	− ± −	− ± −	1.80 ± −	− ± −	− ± −	2.79 ± −	− ± −			
Q2	4.04 ± −	4.44 ± 0.19	4.03 ± 0	4.31 ± 0.36	4.09 ± 0.17	4.20 ± 0	4.04 ± 0.17	− ± −		− ± −	− ± −	4.12 ± −	3.92 ± −	− ± −	− ± −	− ± −	− ± −			
Q3	− ± −	4.84 ± 0.22	4.88 ± 0.30	4.97 ± 0.22	− ± −	5.01 ± −	− ± −	− ± −		− ± −	4.50 ± −	− ± −	4.46 ± −	− ± −	− ± −	− ± −	− ± −			
Q4	5.23 ± 0.09	5.80 ± 0.42	5.52 ± 0.19	6.19 ± 0.51	− ± −	− ± −	− ± −	− ± −		− ± −	4.59 ± −	− ± −	− ± −	− ± −	− ± −	− ± −	− ± −			

Q1 identified the weakest athletes in the bottom 25th percentile, Q2 and Q3 identified athletes within the 25 and 50th and the 50 to 75th percentile, respectively, while Q4 identified the strongest athletes in the > 75th percentile. Sub-junior identifies athletes ≤ 18 years, Junior > 18 ≤ 23 years, Open > 23 ≤ 39 years, Masters 1 > 39 ≤ 49 years, Masters 2 > 49 ≤ 59 years, Masters 3 > 59 ≤ 69 years, and Masters 4 > 69 years. Females and Males athletes are divided in weight categories and expressed in kilograms (Females: 47 kg, 52 kg, 57 kg, 63 kg, 69 kg, 76 kg, 84 kg, and > 84 kg; males: 59 kg, 66 kg, 74 kg, 83 kg, 93 kg, 105 kg, 120 kg, and > 120 kg). “− ± −“ indicates a lack of data to compute the mean ± Standard deviation

The prediction model and predictor coefficients for each lift and total are shown respectively in Table 2a, 2b, 2c, 2d along with Main and Interaction effect p-values.

**Table 2:** **A**: Squat prediction model coefficients for each lift

Squat				
	**Estimate**	**SE**	**tStat**	*p* value
Intercept	-59.28 (-52.95, -65.61)	3.23	-18.36	0.00*
Sex	-0.78 (3.07, -4.63)	1.96	-0.40	0.69
Age	0.45 (0.55, 0.34)	0.05	8.30	0.00*
Body weight	0.65 (0.72, 0.59)	0.03	21.2	0.00*
Initial Strength Level	23.23 (25.68, 20.78)	1.25	18.57	0.00*
Log_time	23.90 (24.81, 22.98)	0.47	51.17	0.00*
Sex*Age	0.18 (0.22, 0.15)	0.02	9.53	0.00*
Sex*Body weight	-0.06 (-0.03, -0.09)	0.01	-4.48	0.00*
Sex*Initial Strength Level	-0.13 (1.04, -1.30)	0.60	-0.21	0.83
Sex*Log_time	-3.25 (-2.96, -3.54)	0.15	-22.26	0.00*
Age*Body Weight	-0.004 (-0.003, -0.005)	0.0004	-11.15	0.00*
Age*Initial strength level	0.001 (0.03, -0.03)	0.02	0.08	0.94
Age*Log_time	-0.19 (-0.18, -0.20)	0.004	-51.72	0.00*
Body weight*Starting strength	0.72 (0.74, 0.69)	0.01	57.89	0.00*
Body weight *Log_time	-0.03 (-0.03, -0.04)	0.003	-10.80	0.00*
Initiual Strength level* Log_time	-4.71 (-4.45, -4.97)	0.13	-35.26	0.00*

SE: Standard Error, * indicates a significant *p* value (< 0.05)

**Table 2:** **B**: Bench press prediction model coefficients for each lift

Bench Press				
	**Estimate**	**SE**	**tStat**	*p* value
Intercept	-31.96 (-28.46, -35.46)	1.79	-17.89	0.00*
Sex	0.44 (2.77, -1.88)	1.19	0.37	0.71
Age	0.41 (0.47, 0.35)	0.03	12.77	0.00*
Body weight	0.25 (0.29, 0.22)	0.02	14.95	0.00*
Initial Strength Level	18.96 (21.05, 16.86)	1.07	17.72	0.00*
Log_time	12.97 (13.47, 12.46)	0.26	50.34	0.00*
Sex*Age	0.02 (0.04, -0.007)	0.01	1.37	0.17
Sex*Body weight	-0.029 (-0.01, -0.05)	0.01	-3.24	0.00*
Sex*Initial Strength Level	1.31 (2.36, 0.26)	0.53	2.44	0.01*
Sex*Log_time	-1.69 (-1.50, -1.88)	0.10	-17.38	0.00*
Age*Body Weight	-0.001 (-0.001, -0.002)	0.0002	-6.94	0.00*
Age*Initial strength level	-0.15 (-0.12, -0.18)	0.02	-9.99	0.00*
Age*Log_time	-0.12 (-0.11, -0.12)	0.002	-56.47	0.00*
Body weight*Starting strength	0.83 (0.85, 0.81)	0.01	79.09	0.00*
Body weight *Log_time	-0.02 (-0.02, -0.02)	0.002	-12.46	0.00*
Initiual Strength level* Log_time	-3.32 (-3.10, -3.53)	0.11	-29.92	0.00*

SE: Standard Error, * indicates a significant *p* value (< 0.05)

**Table 2:** **C**: Deadlift prediction model coefficients for each lift

Deadlift				
	**Estimate**	**SE**	**tStat**	*p* value
Intercept	-46.00 (-38.44, -53.56)	3.86	-11.93	0.00*
Sex	-11.22 (-6.55, -15.88)	2.38	-4.71	0.00*
Age	0.27 (0.42, 0.13)	0.07	3.79	0.00*
Body weight	0.50 (0.56, 0.43)	0.03	14.63	0.00*
Initial Strength Level	16.21 (18.62, 13.80)	1.23	13.19	0.00*
Log_time	32.90 (34.06, 31.74)	0.59	55.70	0.00*
Sex*Age	0.18 (0.23, 0.14)	0.02	8.31	0.00*
Sex*Body weight	0.005 (0.03, -0.03)	0.02	0.30	0.76
Sex*Initial Strength Level	2.63 (3.85, 1.42)	0.62	4.24	0.00*
Sex*Log_time	-4.66 (-4.33, -4.99)	0.17	-27.56	0.00*
Age*Body Weight	-0.002 (-0.001, -0.003)	0.0005	-3.97	0.00*
Age*Initial strength level	-0.01 (0.02, -0.05)	0.02	-0.66	0.51
Age*Log_time	-0.17 (-0.17, -0.18)	0.004	-43.49	0.00*
Body weight*Starting strength	0.80 (0.82, 0.78)	0.01	69.15	0.00*
Body weight *Log_time	-0.11 (-0.10, -0.12)	0.003	-31.77	0.00*
Initiual Strength level* Log_time	-5.40 (-5.12, -5.67)	0.14	-38.47	0.00*

SE: Standard Error, * indicates a significant *p* value (< 0.05)

**Table 2:** **D**: Total prediction model coefficients for each lift

Total				
	**Estimate**	**SE**	**tStat**	*p* value
Intercept	-135.89 (-119.68, -152.09)	8.27	-16.43	0.00*
Sex	-11.33 (-1.24, -21.43)	5.15	-2.20	0.03*
Age	1.46 (1.74, 1.17)	0.15	10.03	0.00*
Body weight	1.25 (1.40, 1.10)	0.08	16.65	0.00*
Initial Strength Level	18.75 (20.98, 16.52)	1.14	16.49	0.00*
Log_time	67.39 (69.78, 64.99)	1.22	55.03	0.00*
Sex*Age	0.30 (0.40, 0.21)	0.05	6.30	0.00*
Sex*Body weight	-0.06 (0.01, -0.12)	0.03	-1.63	0.1
Sex*Initial Strength Level	1.46 (2.57, 0.35)	0.57	2.57	0.01*
Sex*Log_time	-9.30 (-8.57, -10.03)	0.38	-24.80	0.00*
Age*Body Weight	-0.01 (-0.005, -0.009)	0.0009	-8.30	0.00*
Age*Initial strength level	-0.10 (-0.07, -0.13)	0.02	-5.90	0.00*
Age*Log_time	-0.48 (-0.46, -0.49)	0.01	-56.48	0.00*
Body weight*Starting strength	0.81 (0.83, 0.79)	0.01	74.10	0.00*
Body weight *Log_time	-0.16 (-0.14, -0.17)	0.01	-22.43	0.00*
Initiual Strength level* Log_time	-4.39 (-4.14, -4.63)	0.13	-34.82	0.00*

SE: Standard Error, * indicates a significant *p* value (< 0.05)

Prediction model diagnostics and correlation matrix for predictors are reported in supplementary materials (please, see Supplementary materials section). In Table 3, indexes of models’ performance in powerlifting performance prediction across all lifts and Total are displayed.

**Table 3:** Indexes of models’ performance in predicting individual future performances

	MAE (Test)	MSE (Test)	RMSE (Test)	*R*-squared (Test)
Squat	12.00	306.55	17.51	0.91
Bench Press	7.15	108.26	10.41	0.94
Deadlift	12.88	378.27	19.45	0.90
Total	27.73	1642.80	40.53	0.93

**Fig. 2:**
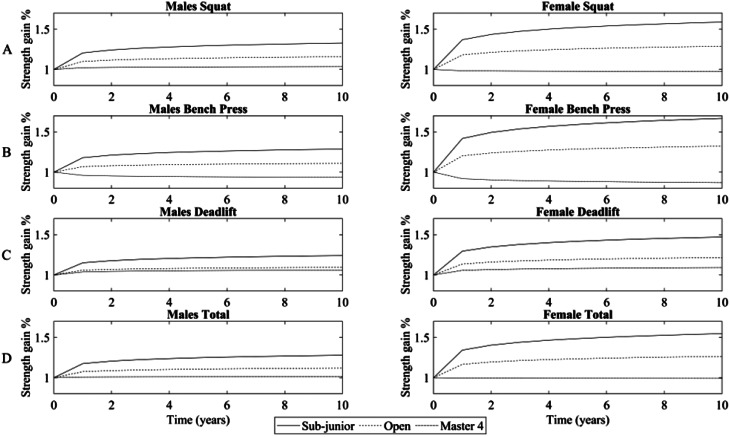
Panel **A** refers to Squat; Panel **B** refers to Bench Press; Panel **C** refers to Deadlift; Panel **D** refers to Total. In the left column of the figure, data relative to males are reported; in the right column of the figure, data relative to females are reported. Age categories’ performance trajectories are plotted over time (i.e., the youngest, the oldest, and Open categories) independently for each lift and sex. Strength gains were rescaled in function of the baseline strength and expressed as a %. The time variable is represented in years to better reflect how performance changes at the beginning of the powerlifting career compared to later on. Age affects the strength changes over time, with the oldest age categories displaying the lowest longitudinal % gain. Moreover, males display lower relative strength gain over time compared to female athletes

Mean Absolute Error (MAE), Mean Squared Error (MSE), Root Mean Squared Error (RMSE), Coefficient of Determination (R-squared);

In Fig. 2, for model visualization purposes, longitudinal strength gains for each lift were rescaled in function of the baseline strength, for both sexes. Moreover, only three age categories were represented to better reflect the age effect on the predictions.

**Fig. 3:**
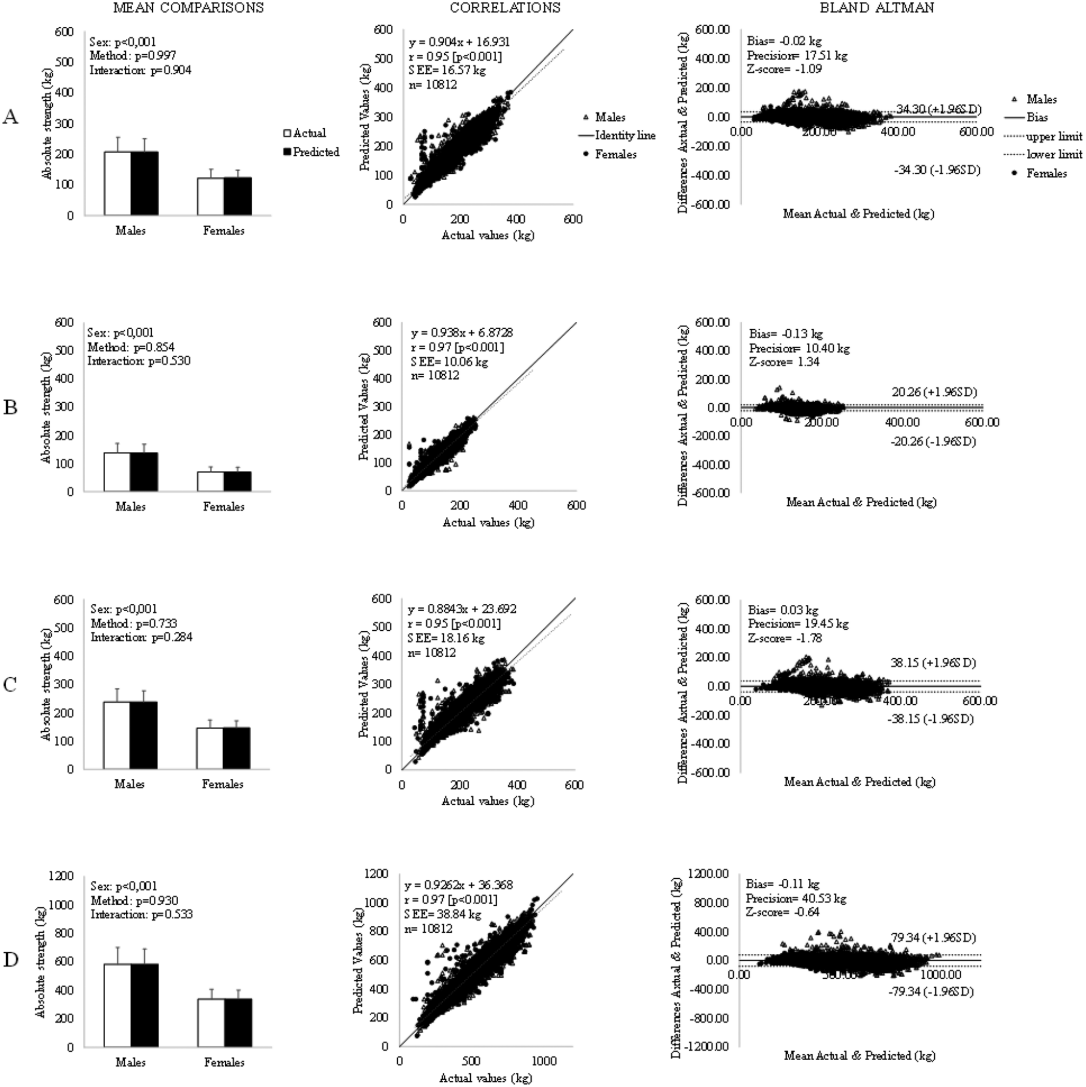
Panel **A** refers to Squat; Panel **B** refers to Bench Press; Panel **C** refers to Deadlift; Panel **D** refers to Total. In the left column of the figure, the comparison between the Actual and Predicted mean values is reported along with the standard deviations (extended lines); in the center column of the figure correlation plots between Actual and Predicted values are shown along with the Pearson correlation coefficient (r), p-value, Standard Error of Estimates (SEE), sample size, regression (dashed line), and identity (solid line) lines. On the right side of the figure, the Bland Altman analysis between Actual and Predicted values is reported: individual differences are plotted as a function of the mean of the two measures. Bias, Precision, and Z-score are shown along with limits of agreement (dashed lines) and bias (solid lines). Δ represents male while ○ represents female powerlifters

The two-way ANOVA showed a main effect of sex, while there was neither a main effect of method nor an interaction effect between actual and predicted values. Since there was no effect of sex on correlations and Bland-Altman analysis between actual and predicted values, Fig. 3 reported the overall sample results while maintaining a graphical distinction between male and female athletes. Correlations between measures were extremely large (0.95 to 0.97), and Bland-Altman analysis showed no significant bias (z-score − 1.78 to − 0.64) with small limits of agreement between actual and predicted values across all lifts. In addition, we provide the model’s performance across different athlete categories (e.g., age groups, weight classes) in supplementary materials (please see Supplementary Materials section) to give more granular insight into the model’s performance in powerlifting performance prediction. Briefly, for each age category, the mean comparisons between methods were not significantly different across all lifts, and the correlations between measures were all large to very large (0.68 to 0.86). Regarding the Bland-Altman analysis, for the Sub-Junior and Junior age categories, a significant bias was found for the Total (Bias: 0.74 kg, Precision: 38.92 kg, z-score: 2.15), while for the Open age category, a significant bias was found for the Squat, Deadlift, and Total (Bias: −0.79 kg, Precision: 18.36 kg, z-score: −2.55; Bias: −1.20 kg, Precision: 20.88 kg, z-score: −4.78; Bias: −2.14 kg, Precision: 42.41 kg, z-score: −3.02). No significant bias was found for Master athletes across all lifts.

Regarding the model’s performance across different weight classes, the mean comparisons between methods were significantly different for Squat, Deadlift, and total in the Lightest weight classes (Females ≤ 52 kg, Males ≤ 66 kg; Squat *p* = 0.023, Deadlift *p* = 0.002; Total *p* = 0.027). The correlations between measures were all large to very large across all lifts and weight classes (0.66 to 0.86). Regarding the Bland-Altman analysis, for the lightest weight categories, a significant bias was found across all lifts (Squat Bias: 2.66 kg, Precision: 13.34 kg, z-score: 7.32, Bench Press Bias: 0.80 kg, Precision: 8.36 kg, z-score: 4.87; Deadlift Bias: 3.44 kg, Precision: 15.50 kg, z-score: 8.01; Total Bias: 6.29 kg, Precision: 31.94 kg, z-score: 7.44) while for the Middleweight categories (Females 57, 63, 69 kg, Males 74, 83, 93 kg), a significant bias was found for the Squat, Deadlift, and Total (Bias: −0.44 kg, Precision: 17.04 kg, z-score: −2.61; Bias: −0.56 kg, Precision: 19.51 kg, z-score: −4.61; Bias: −1.23 kg, Precision: 40.17 kg, z-score: −2.91). No significant bias was found for the Heavyweight categories (Females ≥ 76 kg, Males ≥ 105 kg) across all lifts.

## Discussion

This study aimed to develop and validate a supervised machine-learning model for predicting future performances in European Classic Powerlifters of different sexes, ages, weights, and initial strength levels. A previous predictive model was developed with an “unsupervised” and hardly interpretable (i.e. “Black box”) machine learning method (ELM) for predicting the best score. That model was developed exclusively by using data from male athletes, and the data set included equipped powerlifting data, questioning its application in classic-only powerlifting performance and in female athletes. Based on the analysis of individual historical anthropometric and performance records from a large European dataset of classic powerlifting, a multiple linear regression model was developed and it can effectively predict individual absolute strength in each lift and total. Coaches could use this model for athlete stratification, performance monitoring over time, and for long-term strategic goal-setting. For example, a coach could evaluate if an athlete “seen for the first time” has developed strength optimally or if he/she falls short of their optimal trajectory. Moreover, abrupt deviation from the optimal trajectory over time could represent a warning sign for over-training/nutritional deficiencies/training defects, or, on the contrary, for possible illicit practices. In the case of young athletes, a long-term strategic goal-setting could be performed by predicting their performance over time to identify the competitive level and/or weight category with the highest potential for best positioning.

Moreover, this is the first study to provide a comprehensive (i.e., data relative to Squat, Bench Press, Deadlift, and Total), large (i.e., from 8907 unique lifters), and detailed (i.e., data presented separately for each sex, age, and weight category) description of normative powerlifting performance data in Classic (i.e. without supportive equipment) European athletes. These data could serve coaches to tailor athletes’ training goals and identify individual lift performance deficiencies, athletes’ stratification, and talent identification.

van den Hoek et al. [32] provided comprehensive overall worldwide (Africa, Asia, Europe, North America, South America, and Oceania) powerlifters’ normative data for each lift, using the same publicly available database used in our study. Although powerlifting normative data exist, they refer to the world population, and therefore, those values do not consider the possible differences in powerlifters’ strength levels that may arise from belonging to different geographic areas. Indeed, cultural, social, and economic factors of different geographic areas could affect sports popularity and diffusion, which in turn could affect training practices, competition levels, and discipline developments [26]. Our Classic European normative values are similar to those of Hoek 2024, but this could be because the European sub-cohort represents ~ 34% of the total worldwide dataset, heavily influencing the comprehensive Hoek values (the North American cohort represents ~ 38% while all the other geographic areas represent 26% of the entire database). This result suggests the great diffusion of the powerlifting discipline in Europe and the highly competitive level of European powerlifters globally.

Sex is known to affect strength level, and, in particular, females have less relative strength than men [33]. Our results align with this notion since our female athletes displayed 76%, 64%, 79%, and 75% of the initial strength values compared to men for Squat, Bench press, Deadlift, and Total, respectively.

Aging is known to affect the level of strength. Indeed, Anton et al. [34] showed a ~ 10% difference in maximal strength between open category and older age groups across powerlifters of both sexes. Our results confirm the main effect of age categories on initial strength level, and we found similar age-related losses of relative strength (− 11%,−8%,−10%, and − 11%, for Squat, Bench press, Deadlift, and Total, respectively between the Open and Master 1 categories).

As expected, quartiles also had a main effect on the initial strength level, with stronger athletes (i.e., the athletes within the fourth quartile Q4) having greater relative strength at baseline. This result was expected because it reflects the athlete’s partition in initial strength levels based on their relative strength during their first appearance on the database.

Taking together, the availability of population-specific normative data is essential for contextualizing individual data, talent identification, and facilitating interpretation in support of decision-making for individualized exercise prescription (i.e., level- and goal-specific interventions). In this context, our study offers a large Classic European database of powerlifting performances in competitive powerlifters of different ages, body weights, initial strengths, and sexes.

### Model Training and Validation

This is the first study that attempted to quantitatively predict individual future performances using a multiple linear regression based on a combination of anthropometric (sex and body weight) and personal (age) data, past performances (initial strength), and experience (weeks from the first competition) of powerlifters for each lift (Squat, Bench press, and Deadlift) and Total. All the predictors implemented in the linear model were statistically significant in explaining longitudinal variations in powerlifting relative strength over time (Table 2).

#### Sex

Previous studies [13] showed that sex affects both baseline absolute strength and the rate of longitudinal strength gain, with female athletes displaying less strength but faster strength gains than men, across all lifts. In our study, we found that when considering the absolute values, female athletes display significantly less strength than men for Deadlift and Total, while for Squat and Bench press, sex does not affect the model intercept. Moreover, the model interaction showed that female athletes gain less absolute strength over time than men, having less steep slopes. However, when rescaling longitudinal strength gain as a percentage of baseline absolute strength (Fig. 2), female athletes displayed steeper slopes than males, according to what was found by Latella et al. [13]. For example, a female powerlifter who lifts 100 kg in Bench Press could gain 5 kg over time, representing 10% of the baseline strength, while a male lifting 150 kg could gain 7 kg, representing 4.7% of his baseline strength. These results suggest that female athletes seem more sensible to strength training than males, and this could be partially related to the lowest female strength values displayed at baseline.

#### Age

Latella et al. [13] found that age negatively affects model intercept and model slope, with male Master 4 athletes (with an age > 69 years) displaying the lowest strength level and a negative slope of longitudinal strength, indicating a loss of strength over time. Our results are in accordance with these findings. Indeed, athletes participating in the open category had the greatest absolute strength at baseline, while the Master 4 category had the lowest. Moreover, age also affects the model slope, causing older athletes to have an attenuated magnitude of longitudinal strength compared to younger athletes. Again, rescaling the longitudinal strength gain as a percentage of absolute strength at baseline returns this information more clearly (Fig. 2). Interestingly, in our study male Master 4 displayed a loss in performance over time only in the Bench Press, while female Master 4 powerlifters displayed a strength loss in Squat and Bench press exercises. Although Master powerlifters displayed a loss in muscle strength, their magnitude was less than that related to the aging process for healthy sedentary older adults (~ 0.1 to 1.5% vs. 2.0–5.0% strength loss per year [35]. This difference could be attributed to the protective effect of strength training on the age-related loss of muscle strength that accompanies even normal aging. Taking together, our results gave information about older strength-trained specialized individuals’ adaptation to strength training.

#### Body Weight

Absolute strength is known to be related to body weight, with individuals who have greater body weight displaying greater absolute strength levels; on the contrary, when the absolute strength is normalized by the body mass, there is a decrease in relative strength with increasing body weight [36, 37]. Our results confirm these findings. Moreover, Latella et al. [13], showed that body weight interacts with time, showing a slower rate of strength gains in the heavyweight classes. Our results confirm this finding. In particular, the Deadlift was the lift most affected by bodyweight and time interaction, with a four-fold reduced strength gain compared to the Squat and Bench press (− 0.11 vs. − 0.03 to − 0.02). From a speculative standpoint, increasing the body mass could help reduce the lifting range of motion and improve performance but this advantage is still valid until it allows the execution of the lifting movements properly. For example, in the Deadlift, larger body masses could oppose the reaching of the proper starting position, resulting in a loss of performance. Indeed, it is common for the super heavyweight class (− 120 and 120 kg) to display more strength in Squats than in the Deadlifts. This phenomenon could only explain why the Deadlift is more affected by the body weight and time interaction than the Squat and Bench press since the diminishing relative strength with the increasing body weight also occurs in these lifts. The physiological reason behind this phenomenon is unknown, and even if this phenomenon suggests an upper limit in maximal relative and absolute strength for each body weight, future research is needed [13].

Interestingly, female lifters in the maximum weight classes (− 84 and 84 kg) seem to be the exception, improving their performance over time as the lighter weight classes do. The lower sample of female athletes competing in these weight classes could affect the overall competitive level. Indeed, these athletes displayed lower initial strength levels and lower absolute strength than the lighter-weight classes. Therefore, our results could suggest that these female athletes could gain more strength over time since their strength levels are far from their maximum potential.

#### Initial Strength

As expected, the initial strength level affected the model intercept, with stronger athletes having greater relative strength at baseline. This result was expected because it reflects the athlete’s partition in initial strength levels based on their relative strength during their first appearance on the database.

Baseline strength at the beginning of the powerlifting career is a factor that appears to affect the rate of long-term strength adaptation [3, 14], with the strongest athletes having the slowest longitudinal strength gain. In particular, it was shown that the strongest males displayed a lower longitudinal strength improvement rate than the least strong males, while no differences were observed across initial strength levels for females [27]. Interestingly, our results confirmed this loss in strength gain rate with increasing initial strength level even in female athletes, suggesting a ceiling effect in strength development.

#### Time

Previous studies showed that performance increases with time, and most strength gain occurs within the first year of competition (~ 7.5–12.5% strength increase in the first year, and up to ~ 20% above baseline after 10 years) [13, 24]. Our results confirm these notions since performance showed to increase over time, with a slightly greater rate of improvement occurring in the Squat (a mean increment of 18% after ten years independent of sex, age, and weight categories) compared to Bench Press and Deadlift (a mean increment of 16% after ten years independently of sex, age, and weight categories). Moreover, our results confirmed that the greatest gain in strength occurs within the first year to then slowly reduces its magnitude until reaching a plateau in the following years (10 to 12% in the first year, and down to 0.27 to 0.32% in the tenth year) suggest a diminishing return in strength gain related the augmenting of specialization in strength training [24].

### Model Performance

Only one previous study investigated the validity of a machine learning approach to predict future performances. Chau [20] used an unsupervised method of machine learning called the Extreme Learning Machine (ELM) algorithm; despite its fast training speed and high analysis accuracy, its development was limited to male athletes only and may be hard to interpret for the inclusion of equipped powerlifting data. Compared to the only available previous study, our data provide a fully interpretable model with a better data fit (R^2^ 0.79 vs. R2 from 0.90 to 0.94; root-mean-square error of prediction value of 16.73 vs. RMSE from 10.41 to 19.45 for the lifts and 40.53 for the Total), within the homogeneous context of European, classic, IPF athletes. Moreover, our predictive model could be interpreted easily since it gives precious information about the effect of each predictor on the overall powerlifting performance and individual lifts. Finally, we examined the model prediction validity, accuracy, and precision by comparing predicted and actual values of powerlifting performance. We found non-significant differences between actual and predicted means, extremely large correlations, and a non-significant bias with small limits of agreement across all lifts in the whole sample. The more in-depth investigation of the model’s performance across different age and weight categories showed a statistically significant bias for certain athlete categories (please see supplementary materials). Despite that, the relatively small absolute values of the biases (from − 2.14 kg to 6.29 kg) could be considered negligible under real coaching conditions. A direct comparison of the prediction’s performance with the model proposed by Chau et al. is not possible because the author did not report a comparison of mean, correlation, or bias analysis between predicted and actual values.

## Limitations

Our prediction model is based on simple and accessible data, which can also represent a limitation since it does not consider other important factors that would have contributed to individual performance [13]. Among them, it is known that some anthropometric factors are strictly related to maximal strength (i.e., lean muscle mass or limb circumferences) [8–10]. Moreover, individual training history data, such as past injuries, training modalities, tapering strategies, and the individual chronic training load, are all important factors that could have affected the performance recorded in the dataset but that are all blinded to the model.

As it is known, linear regression is strictly related to the data pool on which is based. In this case, the numerosity of the sample in the lowermost and uppermost weight categories and youngest and oldest age categories are very few, affecting the model prediction performances for these categories. Indeed, the Q-Q plot of residuals of the prediction showed some deviation from the normality of distribution at the tails (please, see Supplementary materials). Despite that, the significant biases found in these age and weight categories could be considered irrelevant under real-life conditions since the magnitude of the values is very small.

There may be a source of model overestimation about the long-term improvements because, generally, the athletes who compete for the longest time are probably the athletes who continue to win and/or improve over time, and consequently, the model does not consider athletes with different performance trajectories.

We limited our dataset to only classic European powerlifters, which could impact the model generalizability, especially if applied to data concerning athletes from different countries (which may have different powerlifting popularity and diffusion and, therefore, different competition levels). Despite that, our mean strength absolute and relative values were similar to what was found in previous studies [32]. Therefore, our model could be able to maintain its validity for powerlifting performance prediction in datasets from different countries. Our dataset did not include data regarding the equipped powerlifting. Although the strength level and normative values for classic and equipped powerlifting are different and not comparable, it could be argued that maybe the long-term strength adaptation and development for the different weight, age, and sex categories do not differ between disciplines and, therefore, our model could be used as well by trainers and coaches for individualizing training goals and benchmarking athlete performance. Finally, our dataset did not include data regarding the non-athlete population. Although the long-term strength adaptation trajectories could be expected to be steeper in recreational or sedentary individuals, the effect of age, sex, and body weight on strength level and strength adaptation trajectory could be the same.

We want to highlight that the decisions made by a machine learning method reflect and are therefore biased by the initial data on which the model is trained. If the dataset contains some selection biases, the resulting predictions can perpetuate these biases. Therefore, we encourage coaches and practitioners to use machine learning models to support a decision-making process rather than use these models blindly.

## Future Developments

Future developments in prediction models should consider collecting and incorporating different data regarding anthropometric and training history data (past injuries, training modalities, tapering strategies, and the amount of training an athlete undergoes before competitions). Other studies could explore alternative machine learning models, expand the dataset to include other regions or powerlifting types, and test the model’s utility in real-world coaching settings. Despite this, it is worth noting that the prediction model could be ameliorated with the increasing availability of data. Indeed, the continuous longitudinal collection of the powerlifting data could serve as a new source of information from which the prediction model could train its prediction ability.

## Conclusions

Our results added information about how strength development trajectories are influenced by age, sex, body weight, and training experience. By taking advantage of large publicly available powerlifting data set records, it was possible to develop an accurate model for predicting future powerlifting performance that could support trainers and coaches in individualizing training goals and benchmarking athlete performance. Moreover, the large normative data provided for each age and weight category could support athletes and coaches in detecting individual lift deficiencies or be used for talent identification and stratification. Finally, we argue that these data provide more important information that goes beyond the scope of the study’s aim, particularly regarding the protective effect against the age-related decline in muscle strength in older individuals, peak strength development in youth, and the involvement of females individuals in strength and power disciplines. This information could be used to sensitize the health benefits of strength training in youth and aging and break down cultural and social barriers to the involvement of female individuals in strength sports disciplines.

To provide a useful tool that uses the predictive model developed in this article, the interested reader is encouraged to explore and utilize a downloadable tool available in the supplemental material. The tool is designed as a spreadsheet to predict future performance in powerlifting.

## Supplementary Information

Below is the link to the electronic supplementary material.

Supplementary Material 1

Supplementary Material 2

Supplementary Material 3

Supplementary Material 4

Supplementary Material 5

Supplementary Material 6

## Data Availability

The datasets analyzed during the current study are available at https://gitlab.com/openpowerlifting/opl-data.

## Abbreviations

ELM: Extreme Learning Machine
PL: Powerlifting
IPF: International Powerlifting Federation
Log_time: Natural log-transformation of time from the first competition
Q: Quartiles
MAE: Mean Absolute Error
MSE: Mean Squared Error
RMSE: Root Mean Squared Error
R-squared: Coefficient of Determination
LOA: Limits of agreement
R: Coefficient
SE: Standard Error
SEE: Standard Error of Estimates
