# Supplementary material for: Predicting Future Performance in Powerlifting: A Machine Learning Approach
Source: Sports Med Open. 2025 Oct 1;11:112. doi: 10.1186/s40798-025-00903-z (PMC12488546; doi:10.1186/s40798-025-00903-z)
Supplement: Supplementary file 1 — Supplementary Material 1 [file 40798_2025_903_MOESM1_ESM.docx]

| Correlation Matrix | | | | | | | | | | | |
| --- | --- | --- | --- | --- | --- | --- | --- | --- | --- | --- | --- |
|  | |  | | **Age** | | **Bodyweight** | | **Initial Strength** | | **Log_time** | |
| Age |  | Pearson's r |  | — |  |  |  |  |  |  |  |
|  |  | df |  | — |  |  |  |  |  |  |  |
|  |  | p-value |  | — |  |  |  |  |  |  |  |
| Bodyweight |  | Pearson's r |  | 0.079 |  | — |  |  |  |  |  |
|  |  | df |  | 54062 |  | — |  |  |  |  |  |
|  |  | p-value |  | < .001 |  | — |  |  |  |  |  |
| Initial Strength |  | Pearson's r |  | -0.154 |  | 0.046 |  | — |  |  |  |
|  |  | df |  | 54062 |  | 54062 |  | — |  |  |  |
|  |  | p-value |  | < .001 |  | < .001 |  | — |  |  |  |
| Log_time |  | Pearson's r |  | 0.223 |  | 0.096 |  | 0.031 |  | — |  |
|  |  | df |  | 54062 |  | 54062 |  | 54062 |  | — |  |
|  |  | p-value |  | < .001 |  | < .001 |  | < .001 |  | — |  |
|  | | | | | | | | | | | |
