# Supplementary material for: Predicting Future Performance in Powerlifting: A Machine Learning Approach
Source: Sports Med Open. 2025 Oct 1;11:112. doi: 10.1186/s40798-025-00903-z (PMC12488546; doi:10.1186/s40798-025-00903-z)
Supplement: Supplementary file 3 — Supplementary Material 3 [file 40798_2025_903_MOESM3_ESM.docx]

Predicting future performance in powerlifting: a machine learning approach.

Sports Medicine - Open

Luca Ferrari^1,2^, Gianluca Bochicchio^1^, Alberto Bottari^1^, Francesco Lucertini^2^, Silvia Pogliaghi^1,3^

^1^University of Verona, Department of Neurosciences, Biomedicine and Movement Sciences, 37131 Verona, Italy;

^2^University of Urbino, Department of Biomolecular Sciences, 61029 Urbino, Italy;

^3^University of Western Ontario, Research Associate Canadian Center for Activity and Ageing, ON N6A 3K7, London, Canada

* Corresponding author: Silvia Pogliaghi, via Felice Casorati, 43, 37131, Verona, Italy; Tel.: +39-045-8425128; [silvia.pogliaghi@univr.it](mailto:silvia.pogliaghi@univr.it)

Luca Ferrari: 0000-0002-9855-9659

Gianluca Bochicchio: 0000-0002-9575-9209

Alberto Bottari: 0009-0002-2869-7324

Francesco Lucertini: 0000-0003-3134-4511

Silvia Pogliaghi: 0000-0002-4394-8550

Fig.2a: Model's performance for Sub Junior and Junior age categories


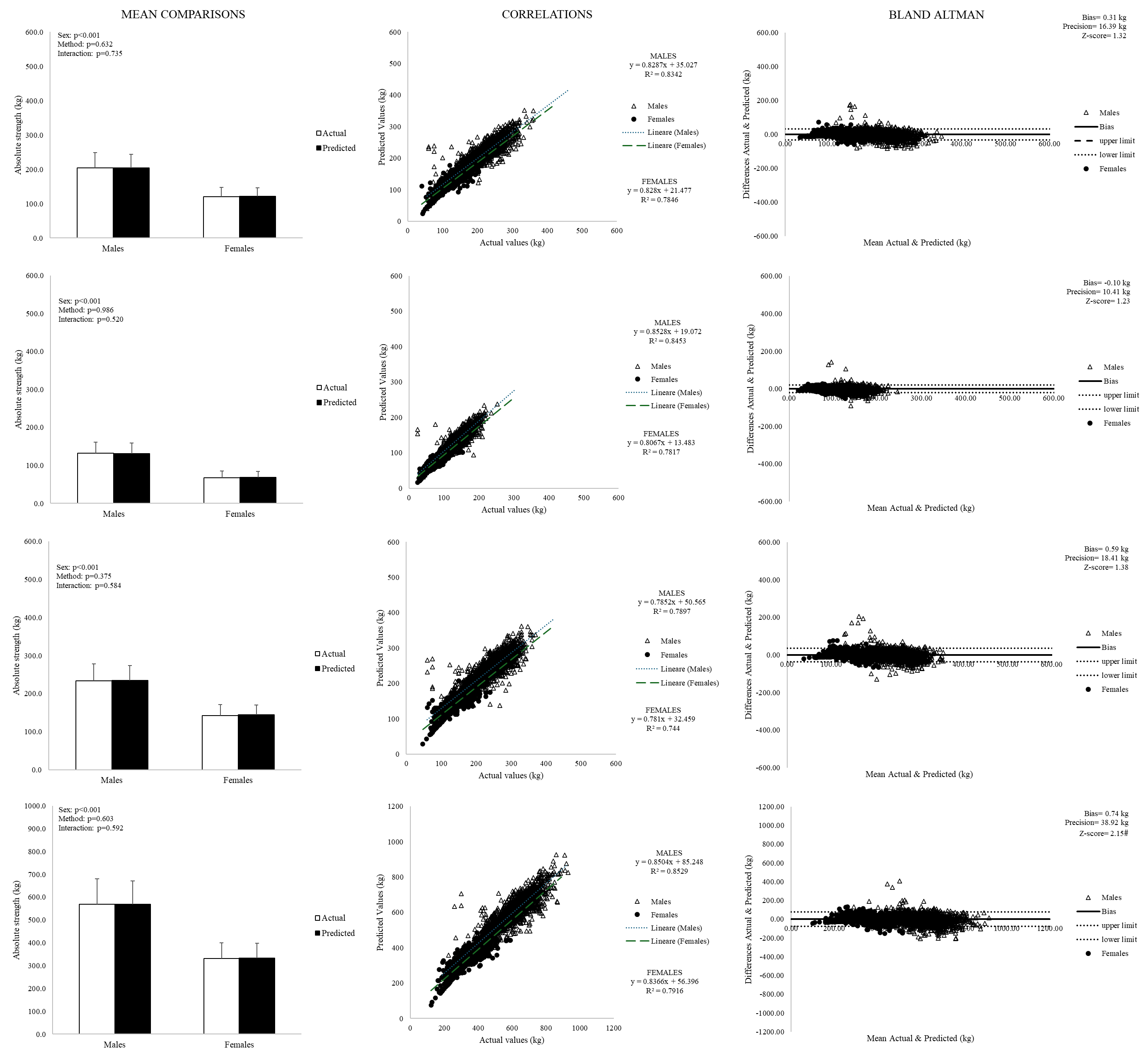


Fig.2a: Panel A refers to Squat; Panel B refers to Bench Press; Panel C refers to Deadlift; Panel D refers to Total. In the left column of the figure, the comparison between the Actual and Predicted mean values is reported; in the center column of the figure, correlation plots between Actual and Predicted values are shown along with the Pearson correlation coefficient (r), p-value, Standard Error of Estimates (SEE), sample size, regression (dashed line), and identity (solid line) lines. On the right side of the figure, the Bland Altman analysis between Actual and Predicted values is reported: individual differences are plotted as a function of the mean of the two measures. Bias, Precision, and Z-score are shown along with limits of agreement (dashed lines) and bias (solid lines). Δ represents male while ○ represents female powerlifters. * indicates significant difference between methods; # indicates a significant Bias.

Fig.2b: Model's performance for Open age categories


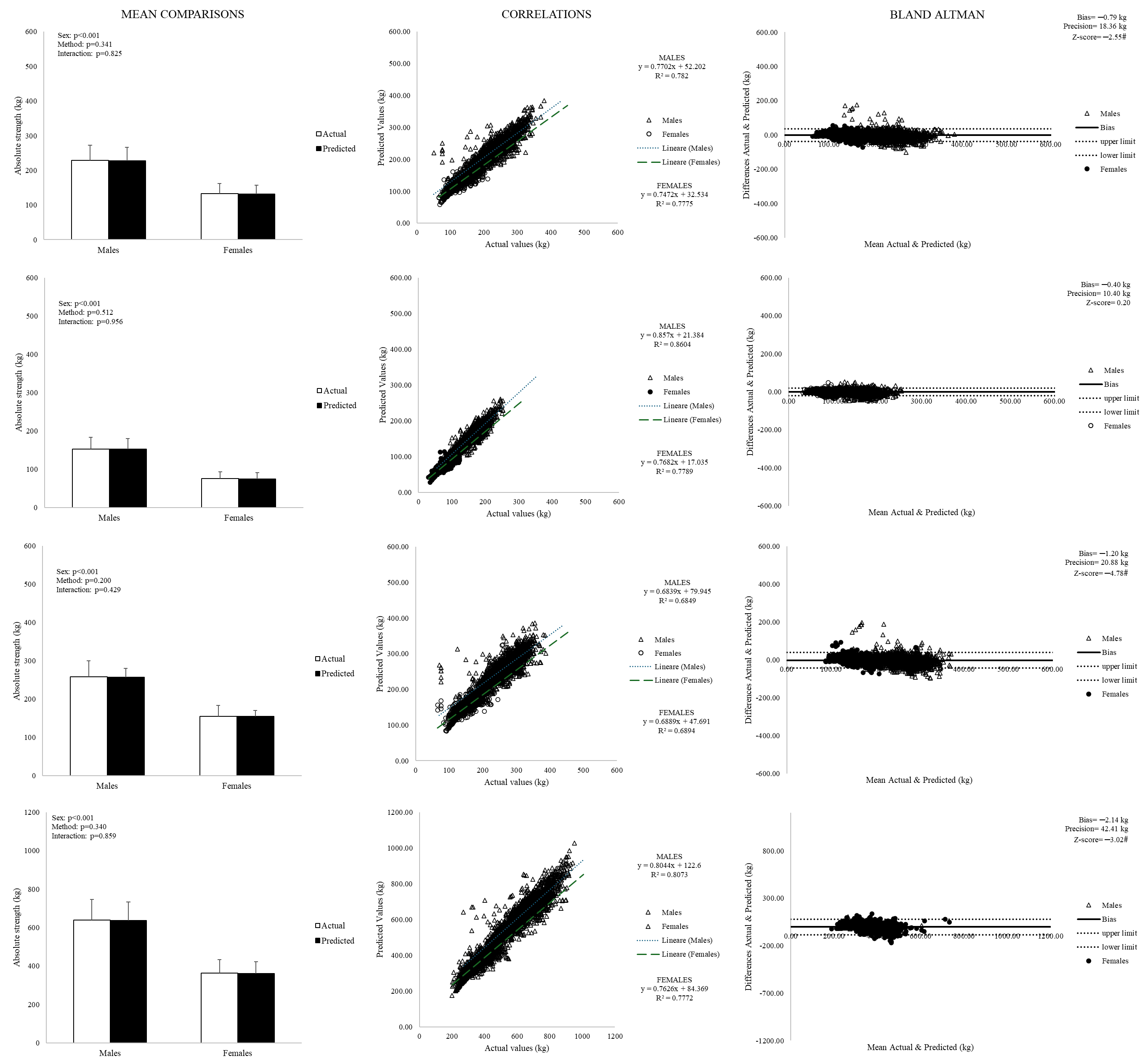


Fig.2b: Panel A refers to Squat; Panel B refers to Bench Press; Panel C refers to Deadlift; Panel D refers to Total. In the left column of the figure, the comparison between the Actual and Predicted mean values is reported; in the center column of the figure, correlation plots between Actual and Predicted values are shown along with the Pearson correlation coefficient (r), p-value, Standard Error of Estimates (SEE), sample size, regression (dashed line), and identity (solid line) lines. On the right side of the figure, the Bland Altman analysis between Actual and Predicted values is reported: individual differences are plotted as a function of the mean of the two measures. Bias, Precision, and Z-score are shown along with limits of agreement (dashed lines) and bias (solid lines). Δ represents male while ○ represents female powerlifters. * indicates significant difference between methods; # indicates a significant Bias.

Fig.2c: Model's performance for Master age categories


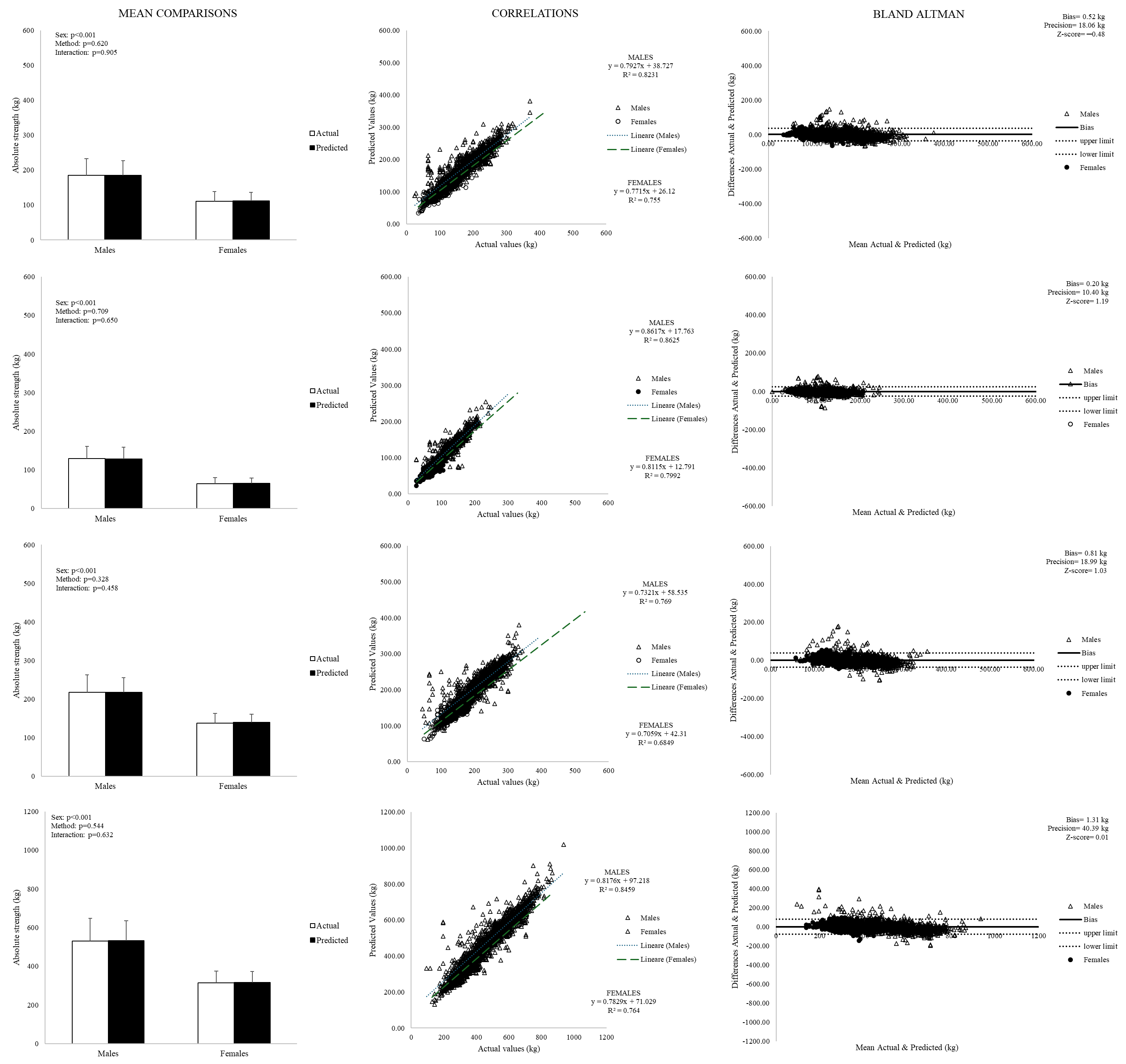


Fig.2c: Panel A refers to Squat; Panel B refers to Bench Press; Panel C refers to Deadlift; Panel D refers to Total. In the left column of the figure, the comparison between the Actual and Predicted mean values is reported; in the center column of the figure, correlation plots between Actual and Predicted values are shown along with the Pearson correlation coefficient (r), p-value, Standard Error of Estimates (SEE), sample size, regression (dashed line), and identity (solid line) lines. On the right side of the figure, the Bland Altman analysis between Actual and Predicted values is reported: individual differences are plotted as a function of the mean of the two measures. Bias, Precision, and Z-score are shown along with limits of agreement (dashed lines) and bias (solid lines). Δ represents male while ○ represents female powerlifters. * indicates significant difference between methods; # indicates a significant Bias.

Fig.2d: Model's performance for Lightweight categories


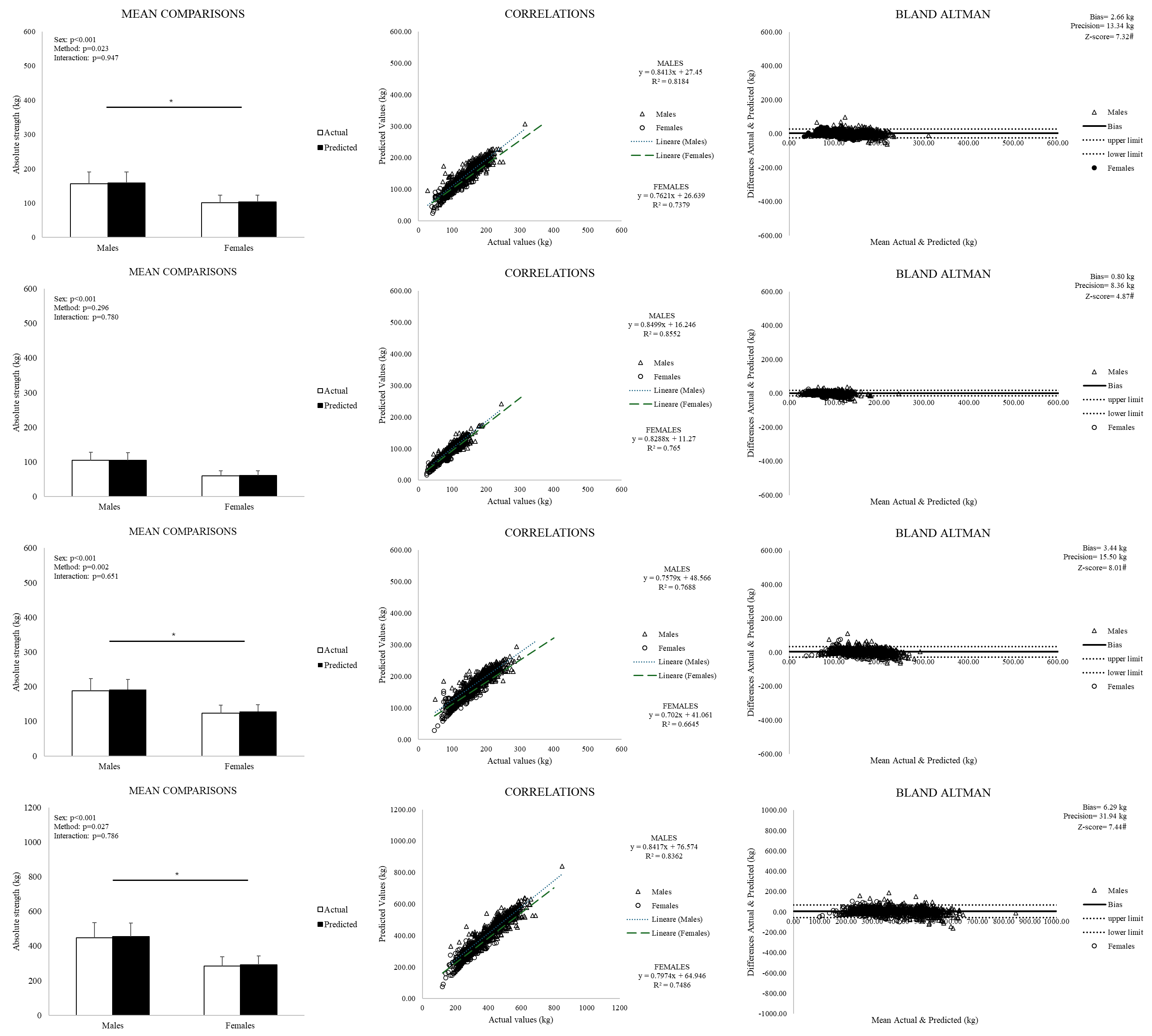


Fig.2d: Panel A refers to Squat; Panel B refers to Bench Press; Panel C refers to Deadlift; Panel D refers to Total. In the left column of the figure, the comparison between the Actual and Predicted mean values is reported; in the center column of the figure, correlation plots between Actual and Predicted values are shown along with the Pearson correlation coefficient (r), p-value, Standard Error of Estimates (SEE), sample size, regression (dashed line), and identity (solid line) lines. On the right side of the figure, the Bland Altman analysis between Actual and Predicted values is reported: individual differences are plotted as a function of the mean of the two measures. Bias, Precision, and Z-score are shown along with limits of agreement (dashed lines) and bias (solid lines). Δ represents male while ○ represents female powerlifters. * indicates significant difference between methods; # indicates a significant Bias.

Fig.2e: Model's performance for Middleweight categories


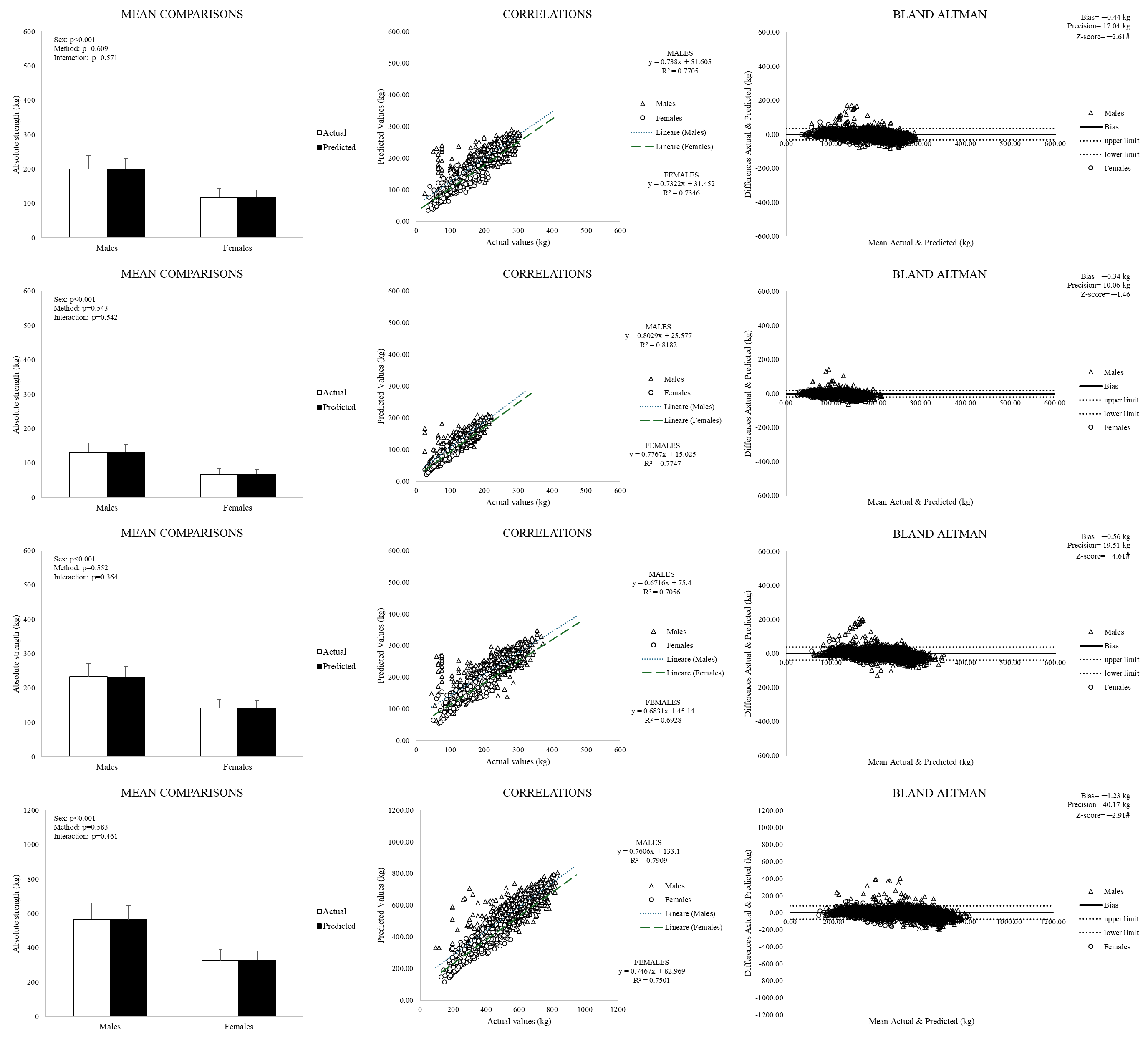


Fig.2e: Panel A refers to Squat; Panel B refers to Bench Press; Panel C refers to Deadlift; Panel D refers to Total. In the left column of the figure, the comparison between the Actual and Predicted mean values is reported; in the center column of the figure, correlation plots between Actual and Predicted values are shown along with the Pearson correlation coefficient (r), p-value, Standard Error of Estimates (SEE), sample size, regression (dashed line), and identity (solid line) lines. On the right side of the figure, the Bland Altman analysis between Actual and Predicted values is reported: individual differences are plotted as a function of the mean of the two measures. Bias, Precision, and Z-score are shown along with limits of agreement (dashed lines) and bias (solid lines). Δ represents male while ○ represents female powerlifters. * indicates significant difference between methods; # indicates a significant Bias.

Fig.2f: Model's performance for Heavyweight categories


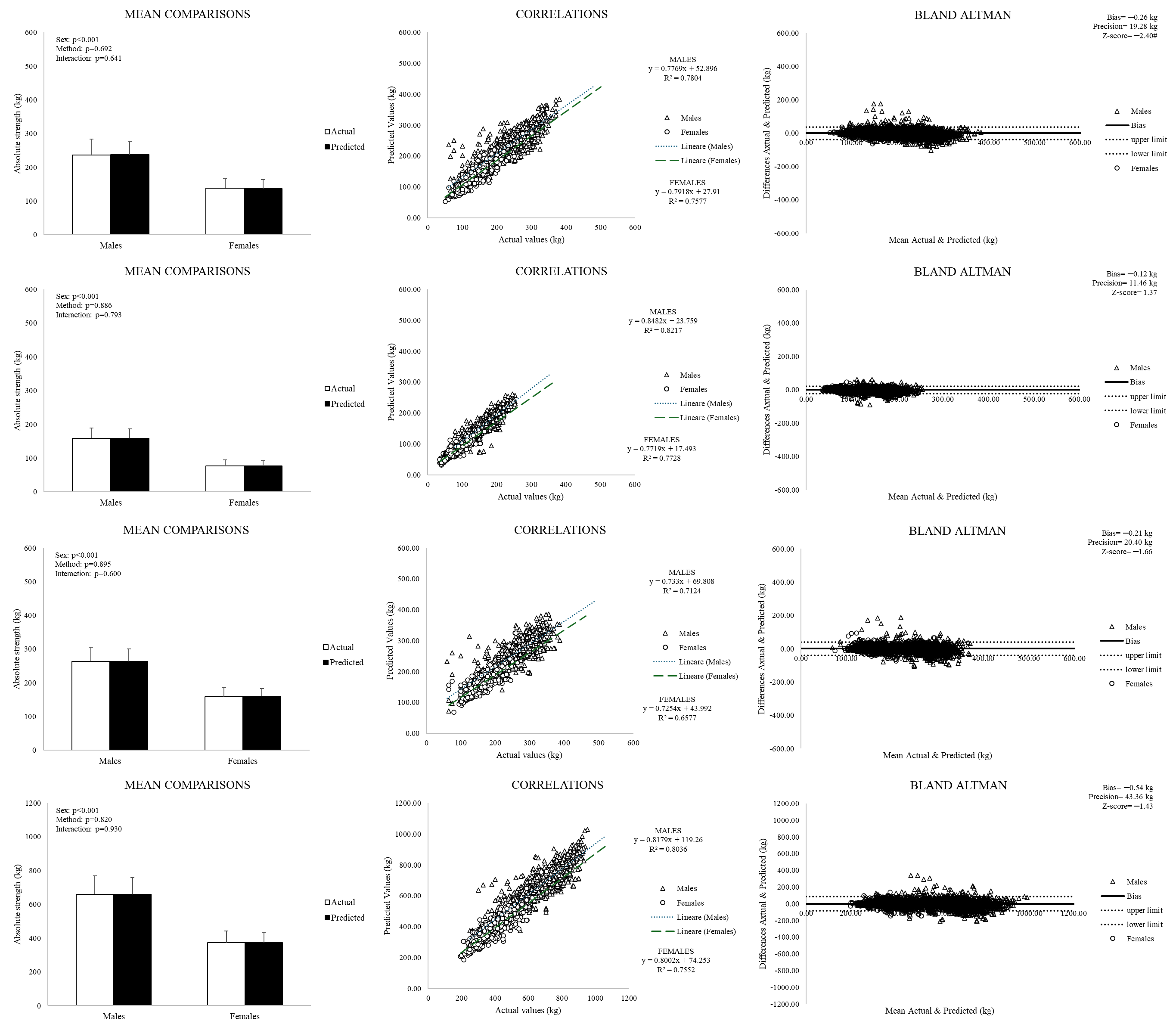


Fig.2f: Panel A refers to Squat; Panel B refers to Bench Press; Panel C refers to Deadlift; Panel D refers to Total. In the left column of the figure, the comparison between the Actual and Predicted mean values is reported; in the center column of the figure, correlation plots between Actual and Predicted values are shown along with the Pearson correlation coefficient (r), p-value, Standard Error of Estimates (SEE), sample size, regression (dashed line), and identity (solid line) lines. On the right side of the figure, the Bland Altman analysis between Actual and Predicted values is reported: individual differences are plotted as a function of the mean of the two measures. Bias, Precision, and Z-score are shown along with limits of agreement (dashed lines) and bias (solid lines). Δ represents male while ○ represents female powerlifters. * indicates significant difference between methods; # indicates a significant Bias.
