# Supplementary material for: Predicting Future Performance in Powerlifting: A Machine Learning Approach
Source: Sports Med Open. 2025 Oct 1;11:112. doi: 10.1186/s40798-025-00903-z (PMC12488546; doi:10.1186/s40798-025-00903-z)
Supplement: Supplementary file 4 — Supplementary Material 4 [file 40798_2025_903_MOESM4_ESM.docx]

Predicting future performance in powerlifting: a machine learning approach.

Sports Medicine - Open

Luca Ferrari^1,2^, Gianluca Bochicchio^1^, Alberto Bottari^1^, Francesco Lucertini^2^, Silvia Pogliaghi^1,3^

^1^University of Verona, Department of Neurosciences, Biomedicine and Movement Sciences, 37131 Verona, Italy;

^2^University of Urbino, Department of Biomolecular Sciences, 61029 Urbino, Italy;

^3^University of Western Ontario, Research Associate Canadian Center for Activity and Ageing, ON N6A 3K7, London, Canada

* Corresponding author: Silvia Pogliaghi, via Felice Casorati, 43, 37131, Verona, Italy; Tel.: +39-045-8425128; [silvia.pogliaghi@univr.it](mailto:silvia.pogliaghi@univr.it)

Luca Ferrari: 0000-0002-9855-9659

Gianluca Bochicchio: 0000-0002-9575-9209

Alberto Bottari: 0009-0002-2869-7324

Francesco Lucertini: 0000-0003-3134-4511

Silvia Pogliaghi: 0000-0002-4394-8550

| Table 4: comparison of different machine learning models on a random sub-sample of 100 athletes | | | | |
| --- | --- | --- | --- | --- |
|  | MAE (Test) | MSE (Test) | RMSE (Test) | RSquared (Test) |
| Tree | 73.82 | 10050.00 | 100.25 | -0.29 |
| Ensemble | 58.65 | 4232.80 | 65.06 | 0.45 |
| Linear regression | 23.10 | 875.04 | 29.58 | 0.89 |
| Mean Absolute Error (MAE), Mean Squared Error (MSE), Root Mean Squared Error (RMSE), Coefficient of Determination (R-squared); | | | | |
